# Dual-acting stapled peptides target both HIV-1 entry and assembly

**DOI:** 10.1186/1742-4690-10-136

**Published:** 2013-11-15

**Authors:** Hongtao Zhang, Francesca Curreli, Abdul A Waheed, Peter Y Mercredi, Mansi Mehta, Pallavi Bhargava, Daniel Scacalossi, Xiaohe Tong, Shawn Lee, Alan Cooper, Michael F Summers, Eric O Freed, Asim K Debnath

**Affiliations:** 1 Laboratory of Molecular Modeling, Drug Design, Lindsley F. Kimball Research Institute of the New York Blood Center, 310 E 67th Street, New York, NY 10065, USA; 2 Virus-Cell Interaction Section, HIV Drug Resistance Program, National Cancer Institute-Frederick, Frederick, MD 21702, USA; 3 Howard Hughes Medical Institute and Department of Chemistry and Biochemistry, University of Maryland, Baltimore County, 1000 Hilltop Circle, Baltimore, MD 21250, USA; 4 CPC Scientific, Inc., 1245 Reamwood Ave., Sunnyvale, CA 94089, USA; 5 School of Chemistry, Joseph Black Building, University of Glasgow, Glasgow G12 8QQ, Scotland, UK

**Keywords:** HIV-1, Capsid, Virus assembly, Virus entry, Stapled peptides, NMR, SPR, ITC, Drug-resistance

## Abstract

**Background:**

Previously, we reported the conversion of the 12-mer linear and cell-impermeable peptide CAI to a cell-penetrating peptide NYAD-1 by using an *i,i + 4* hydrocarbon stapling technique and confirmed its binding to the C-terminal domain (CTD) of the HIV-1 capsid (CA) protein with an improved affinity (K_d_ ~ 1 μM) compared to CAI (K_d_ ~ 15 μM). NYAD-1 disrupts the formation of both immature- and mature-like virus particles in in vitro and cell-based assembly assays. In addition, it displays potent anti-HIV-1 activity in cell culture against a range of laboratory-adapted and primary HIV-1 isolates.

**Results:**

In this report, we expanded the study to *i,i + 7* hydrocarbon-stapled peptides to delineate their mechanism of action and antiviral activity. We identified three potent inhibitors, NYAD-36, -66 and -67, which showed strong binding to CA in NMR and isothermal titration calorimetry (ITC) studies and disrupted the formation of mature-like particles. They showed typical α-helical structures and penetrated cells; however, the cell penetration was not as efficient as observed with the *i,i + 4* peptides. Unlike NYAD-1, the *i,i + 7* peptides did not have any effect on virus release; however, they impaired Gag precursor processing. HIV-1 particles produced in the presence of these peptides displayed impaired infectivity. Consistent with an effect on virus entry, selection for viral resistance led to the emergence of two mutations in the gp120 subunit of the viral envelope (Env) glycoprotein, V120Q and A327P, located in the conserved region 1 (C1) and the base of the V3 loop, respectively.

**Conclusion:**

The *i,i + 7* stapled peptides derived from CAI unexpectedly target both CA and the V3 loop of gp120. This dual-targeted activity is dependent on their ability to penetrate cells as well as their net charge. This mechanistic revelation will be useful in further modifying these peptides as potent anti-HIV-1 agents.

## Background

Human immunodeficiency virus type 1 (HIV-1) is the etiological agent of acquired immunodeficiency syndrome (AIDS). The AIDS epidemic remains a major health crisis worldwide -- According to the 2012 AIDS Epidemic Update (*UNAIDS*) approximately 34 million people are currently living with HIV/AIDS, which is the sixth leading cause of death worldwide and the third among low-income groups (WHO fact sheets, June 2011). Approximately 30 anti-HIV drugs have been approved by the US FDA, a majority of which target one of the three key viral enzymes: reverse transcriptase (RT), protease (PR), and integrase (IN). The first inhibitor of viral entry, Enfuvirtide, was approved in 2003 [1,2] and another entry inhibitor targeted to the CCR5 receptor, Maraviroc, was approved [3]. The introduction of highly active antiretroviral therapy (HAART) has significantly decreased morbidity and mortality among HIV-1-infected patients with access to these drugs. However, the development of drug resistance and issues related to drug tolerability and compliance often pose a considerable challenge to current therapies [4-8]. These concerns, together with recent reports of failure in clinical trials of HIV vaccines and several microbicides, reinforce the critical need to identify new targets for the development of novel classes of anti-HIV-1 drugs.

The HIV-1 genome is composed of three major genes, *gag*, *pol* and *env.* The *gag* gene encodes the Gag protein, the critical structural protein of HIV-1. The *pol* gene encodes the aforementioned viral enzymes, which are essential for HIV-1 replication. The *env* gene encodes the viral envelope (Env) glycoproteins, which play a critical role in virus entry. Virus assembly is a key step in the HIV-1 life cycle, which occurs through the controlled polymerization of the Gag polyprotein [9-11] to form spherical immature non-infectious virus particles that bud out from the plasma membrane. During or shortly after virus release, the particles undergo a process known as maturation. During this step, the Gag polyprotein precursor is sequentially cleaved by PR to matrix (MA), capsid (CA), nucleocapsid (NC), and p6 domains, as well as two spacer proteins (SP1 and SP2). This process triggers a dramatic change in particle morphology during which the CA protein, liberated from the Gag precursor, reassembles into a conical core that surrounds the viral genome. After the virus enters the cell, the conical core undergoes controlled disassembly concomitant with the conversion of the single-stranded viral RNA genome to double-stranded DNA by RT [12-14]. CA thus plays an important role in both the early and late stage of HIV replication, making it an attractive target for novel anti-HIV drugs [15-22].

In 2005, a 12-mer peptide (CAI), identified by phage-display, was reported to disrupt both immature- and mature-like particles *in vitro* by targeting the C-terminal domain (CTD) of HIV-1 CA [21]. However, it could not inhibit HIV-1 in cell culture due to its lack of cell permeability [23]. Subsequently, we converted CAI to a cell-penetrating peptide (NYAD-1) by using a hydrocarbon stapling technique and confirmed its binding to the CTD [24]. NYAD-1, which is an *i,i + 4* staple peptide, disrupts the formation of both immature- and mature-like particles in cell-free and cell-based assembly systems. In addition, NYAD-1 displays potent anti-HIV-1 activity in cell culture against a range of laboratory-adapted and primary HIV-1 isolates (4.2 – 21 μM). It binds to a hydrophobic pocket, identified previously in x-ray studies of CTD complexed with CAI [25], with an improved affinity (K_d_ ~ 1 μM) compared to CAI (K_d_ ~ 15 μM) [24].

Here we report the mechanism of action and antiviral activity of a series of *i,i + 7* stapled peptides derived from CAI. We show that this class of stapled peptides inhibit both assembly of infectious HIV-1 and its entry; thereby acting as dual-targeted inhibitors. NMR studies indicate that these stapled peptides strongly bind to HIV-1 CA, although not all of them significantly perturb i*n vitro* CA assembly. In addition, the ability of these peptides to inhibit virus assembly appears to be dependent on the efficiency of cell penetration. Resistance studies to delineate the target and mechanism of inhibition suggested the involvement of the gp120 V3 loop, a region of gp120 critical for Env-mediated membrane fusion and viral infection [26,27]. Biophysical studies using isothermal titration calorimetry (ITC) confirmed that these peptides bind strongly to the V3 loop. The critical findings detailed here illustrate dual inhibition of HIV-1 assembly and viral entry through specific targeting of HIV-1 CA and gp120, respectively.

## Results

### *i,i + 7* stapled peptides show antiviral potency in multi-cycle infection assay

We used a multi-cycle infectivity assay as a first-step screening tool to measure the anti-HIV-1 activity of 16 *i,i + 7* stapled peptides using laboratory-adapted, X4-tropic HIV-1 IIIB in MT-2 cells. We measured the inhibition of p24 production in MT-2 cells treated over a range of concentrations of peptides and calculated the concentration required to inhibit 50% of the production of p24 (IC_50_). We also evaluated the cytotoxicity of the stapled peptides by the XTT method [28]. The cytotoxicity assessment was performed in parallel with the HIV-1 inhibition assay. The results (Table 1) indicate that NYAD-36, NYAD-66 and NYAD-67 display the best antiviral activity and inhibit infection by HIV-1 IIIB with low-μM potency (IC_50_ ~ 1.5-3.9 μM). These peptides also have a good selectivity index (SI = CC_50_/IC_50_) ranging from >27-126. We have selected these three peptides for subsequent studies for delineating their mechanism of action.

**Table 1:** **Antiviral activity (IC**_**50**_**) and cytotoxicity (CC**_**50**_**) of *i & i + 7 *** **stapled peptides against HIV-1**_**IIIB **_**in MT-2 cells**

**Peptide**	**Sequence**	**Net charge**	**IC**_**50 **_**(μM ± SD) in MT-2**	**CC**_**50 **_**(μM ± SD) in MT-2**	**SI**
CAI	ITFEDLLDYYGP-amide (Linear reference)	-2	>100	>100	>1.0
NYAD-401	Ac-IRQGPKEPFRDYVDR-amide (Linear control for NMR)	1	>100	>100	>1.0
NYAD-41	Ac-ISFDELLDYYGESGS (linear control based on NYAD-36)	-5	>100	>100	>1.0
NYAD-33	Ac-ISF-**R8**-EWLQYY-**S5**-R-amide	0	3.37 ± 0.06	9.2 ± 0.26	2.7
NYAD-36	Ac-ISF-**R8**-ELLDYY-**S5**-ESGS-amide	-4	1.5 ± 0.17	>189.4	>126
NYAD-43	Ac-ISF-**R8**-ELEDYY-**S5**-ESGS	-5	>53.34	>106.6	>2.0
NYAD-44	Ac-ISF-**R8**-ELLDYY-**S5**-ESGSKKK-amide	0	11.7 ± 1	17.2 ± 1.4	1.5
NYAD-46	Ac-ISF-**R8**-QLLDYY-**S5**-QSGSK-amide	0	5.5 ± 1	10.6 ± 0.6	1.9
NYAD-55	Ac-ISF-**R8**-ELLDYY-**S5**-EKSGSKD-amide	-2	>89.7	>89.7	>1.0
NYAD-56	Ac-ISF-**R8**-ELLRYY-**S5**-R-amide	1	4.8 ± 0.9	16.4 ± 1.2	3.4
NYAD-57	ISF-**R8**-ELLNYY-**S5**-ESGS-amide	-1	8.7 ± 0.7	39.6 ± 4.4	4.5
NYAD-58	IRF-**R8**-QLLNYY-**S5**-ESGS-amide	1	6.9 ± 0.06	14.9 ± 1	2.1
NYAD-61	Ac-RSF-**R8**-RLLDYY-**S5**-ESGS-amide	0	10.2 ± 1.1	31.7 ± 1.4	3.1
NYAD-62	Ac-RRF-**R8**-ELLDYY-**S5**-ESGS-amide	-1	8 ± 0.3	15.4 ± 0.2	1.9
NYAD-63	Ac-R-**R8**-FAELLD-**S5**-YGESGS-amide	-2	57.1 ± 3.2	>113	>2.0
NYAD-64	Ac-SK-**R8**-ELLDYY-**S5**-ESGS-amide	-2	>109	>109	>1.0
NYAD-65	Ac-RSFE-**R8**-LLDYYG-**S5**-SGS-amide	-1	>116	>116	>1.0
NYAD-66	Ac-ISF-**R8**-ELLDYY-**S5**-ED-amide	-4	3.94 ± 0.32	>115 (0%)^a^	>29.1
NYAD-67	Ac-ISF-**R8**-EWLQAY-**S5**-EDE-amide	-4	3.88 ± 0.3	>107.4 (0%)^a^	>27.7

^a^The number in parenthesis indicates % toxicity at that dose; S5 [(S)-2-(4′-pentenyl)alanine] and R8 [(R)-2-(7′-octenyl)alanine] indicate stapling sites in the peptide sequence.

### Hydrocarbon stapling enhances α-helicity of the stapled peptides

**Figure 1:**
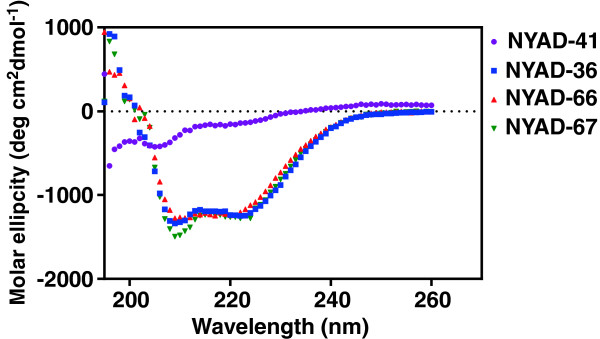
**CD spectra of NYAD-41, NYAD-36, NYAD-66 and NYAD-67 to measure their secondary structures.** CD spectra were measured at 25°C in 1x PBS in the presence of 1%–20% (v/v) acetonitrile at a final concentration of peptide of 100 μM. NYAD-36, NYAD-66 and NYAD-67 showed typical wavelength minima at 208 nm and 222 nm, whereas NYAD-41 showed a minimum at 205 nm.

Previously we have shown that the α-helical characteristics of *i,i + 4* stapled peptides such as NYAD-1 and NYAD-201 is essential for these peptides to penetrate cells in order for them to target HIV-1 CA and inhibit virus assembly and maturation [19,24]. In this study, we also used circular dichroism (CD) to characterize the secondary structure of *i,i + 7* stapled peptides and a linear version of the NYAD-36 in solution, NYAD-41. The stapled peptides showed typical α-helical spectra with minima at 222 and 208 nm; whereas the linear peptide, NYAD-41, did not show these minima, indicative of a random structure in solution (Figure 1). The data confirm that *i,i + 7* hydrocarbon stapling enhances the α-helicity of the linear peptide, NYAD-41, as previously reported [19,24].

### *i,i + 7* Stapled peptides penetrate cells less efficiently than *i,i + 4* stapled peptides

**Figure 2:**
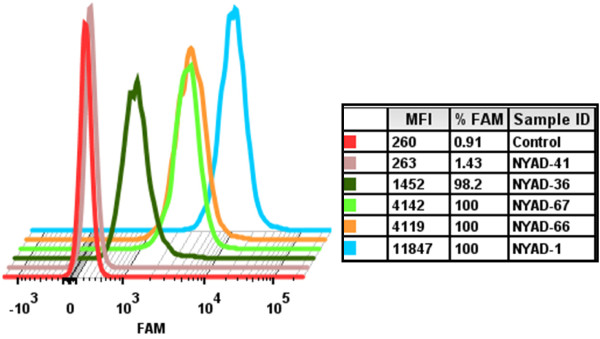
**Cell penetration of staple peptides in 293T cells.** FACS analyses of 293T cells incubated with 4 μM FAM-conjugated peptides for 20 h at 37°C. The cells were treated with 0.25% Trypsin-EDTA for 30 min at 37°C and washed twice with 1x PBS before and after Trypsin-EDTA treatment. The experiment was repeated twice with similar results. The mean fluorescence intensity (MFI) was used to quantify the cellular uptake of FAM-conjugated peptides. NYAD-1 was used as positive control and a FAM-conjugated linear peptide, NYAD-41, was used as a negative control.

With the exception of select positively charged peptides, the electrostatic charge of amino acid side chains and the polarity of the peptide backbone generally impede the transduction of peptides across cellular membranes. Our previous reports demonstrated that *i,i + 4* stapled peptides efficiently penetrate 293T cells. The net charge of the *i,i + 4* stapled peptide NYAD-1 is 0 while the net charge of each of the three selected *i,i + 7* stapled peptides is -4. To test if the -4 net charges would affect the translocation of *i,i + 7* stapled peptides across the plasma membrane, we used confocal microscopy (Additional file 1: Figure S1) and FACS analysis (Figure 2) to study the cell penetration of these peptides. The mean fluorescence intensity (MFI) was used to quantify the cellular uptake of FAM-conjugated peptides. We used NYAD-1 as a control. The data in Additional file 1: Figure S1 and Figure 2 indicate that NYAD-36, NYAD-66 and NYAD-67 apparently penetrate cells less efficiently than NYAD-1 under identical experimental condition. Among *i,i + 7* stapled peptides NYAD-66 and NYAD-67 showed a better penetration profile than NYAD-36.

### *i,i + 7* stapled peptides bind HIV-1 CA

**Figure 3:**
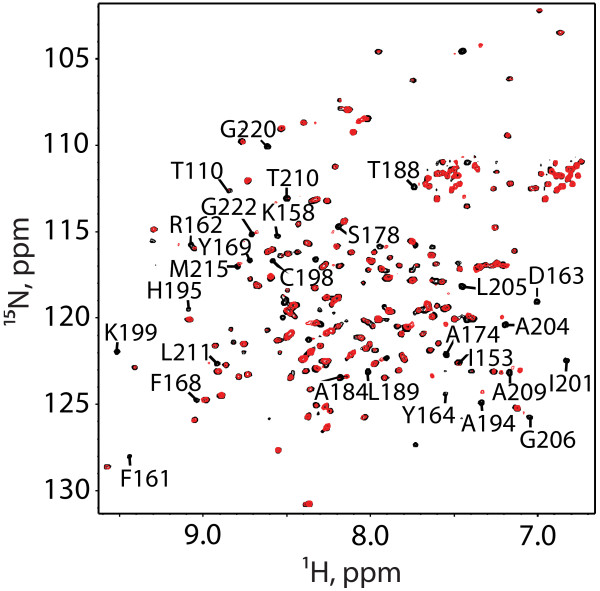
**Overlay of 2D **^**1**^**H-**^**15**^**N HSQC spectra obtained for the HIV-1 capsid monomer (P24-W184A/M185A) protein upon titration with NYAD-36.** Ratios are as follows, 0:1 (Black) and 2:1 (Red), NYAD-36:P24.

^1^H-^15^N heteronuclear single quantum coherence (HSQC) NMR spectra were obtained for a monomeric CA construct containing mutations designed to inhibit CA-CA interactions (W184A/M185A mutations) [29-31]. Spectra were obtained for proteins in the absence and presence of NYAD-36, -66 and -67 peptides and were analyzed using previously reported NMR signal assignments [24,29]. Titrations with stapled peptides NYAD-36, and -66 and -67 resulted in significant NMR spectral changes consistent with tight binding, and all but a few of the affected signals were associated with residues of the CA C-terminal domain, as expected [32]. Representative ^1^H-^15^N HSQC spectra obtained upon titration of NYAD-36 into HIV-1 CA are shown in (Figure 3). The spectral changes are consistent with those observed upon titration of NYAD-1 into the isolated CA-CTD domain [24].

### *i,i + 7* stapled peptides bind to the CA CTD with an affinity similar to that of the *i,i + 4* peptide, NYAD-1

We used isothermal titration calorimetry (ITC) to estimate the binding affinity (expressed as the dissociation constant *K*_d_) of NYAD-36, NYAD-66 and NYAD-67 to the double-mutant version (W184A/M185A) of the CA CTD. The double-mutant CTD was used to avoid CA dimer formation observed with the WT CTD. The stoichiometry (n), affinity (*K*_d_), and thermodynamic parameters such as ΔH and ΔS, were obtained and the data were analysed using MicroCal Origin 7.0 software. The integrated binding isotherms (Additional file 1: Figure S2a-c) were fit to a single-site binding model. The binding affinities of NYAD-36, NYAD-66 and NYAD-67 were 10.12 ± 1.4 μM, 3.60 ± 0.16 μM and 2.63 ± 0.22 μM, respectively. NYAD-1 was used as a control and its binding affinity to CA was 2.00 ± 0.19 μM (Additional file 1: Figure S2d). NYAD-36 showed ~ 3-5-fold lower affinity towards the CA CTD compared to NYAD-1, -66 and -67.

### *i,i + 7* stapled peptides disrupt CA assembly in vitro

**Figure 4:**
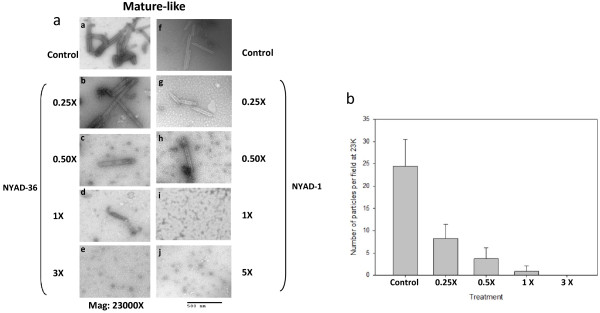
**Inhibition of *in vitro *assembly by NYAD-36.** Negatively stained EM images of mature-like particles resulting from *in vitro* assembly of CA proteins in the presence of **(a)** no peptide (Control), 0.25-, 0.5-, 1.0-, and 3-fold molar equivalent of NYAD-36. and 0.25-, 0.5-, 1.0-, 3.0- and 5-fold molar equivalent of NYAD-1. **(b)** Quantification of mature-like particles resulting from *in vitro* assembly of CA proteins in the presence of no peptide (Control), 0.25-, 0.5-, 1.0-, 3.0-fold molar equivalent of NYAD-36.

It has been demonstrated previously that under appropriate conditions HIV-1 CA will assemble into tubes in which the CA monomers are arranged in a hexameric lattice analogous to that found in the mature, conical core [33-35]. This in vitro assembly reaction is thus useful for examining the effect of CA-binding inhibitors on core assembly. We used this approach to evaluate the effect of NYAD-36, -66 and -67 on CA assembly [19]. Assembly reactions were performed with no peptide (control) or an increasing dose of 0.25- to 3.0-fold molar equivalent of NYAD-36. In the presence of peptide, the CA tubes that formed were either severely damaged or disintegrated (Figure 4a). A clear dose–response effect was observed (Figure 4b). NYAD-1 was used as a control. A similar trend in preventing tube assembly was observed with NYAD-66 and NYAD-67 (data not shown).

**Figure 5:**
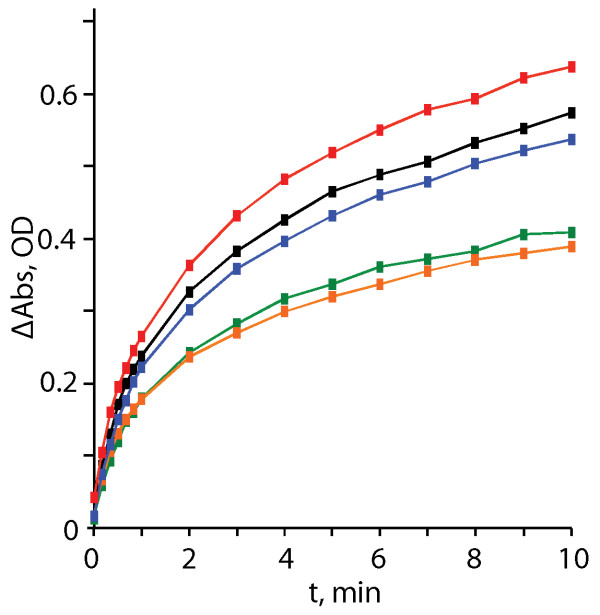
**Turbidity assay results showing the effects of CA-binding to staple peptides on *in vitro *capsid assembly.** Compound: CA ratios and initial rates [millioptical density units (mOD)] per minute, reported as the mean ± standard deviation from three experiments) are as follows: red, 2:1 NYAD-401 (does not bind CA) (223 ± 32.5 mOD/min); black, 1.2uL DMSO control (216 ± 6.23 mOD/min); blue, 2:1 NYAD-67 (205 ± 18.89 mOD/min); green, 2:1 NYAD-66 (166 ± 18.91 mOD/min); orange, 2:1 NYAD-36 (158 ± 24.71 mOD/min).

The ability of stapled peptides to disrupt CA-CA interactions was also assessed using a turbidity-based in vitro CA assembly assay. This assay is based on the observation that the turbidity of a CA-containing solution increases in proportion to the extent of assembly [18,36]. A control peptide comprising the sequence for the capsid major homology region (MHR), NYAD-401, had no observable effect on CA assembly, as expected. Compounds NYAD-36 and NYAD-66 both reduced the extent of CA assembly in a dose-dependent manner, exhibiting maximal effect at a CA:peptide ratio of 1:1 (Figure 5). Interestingly, although NMR studies clearly show that NYAD-67 binds tightly to CA, the binding did not influence the rate or extent of CA assembly in this *in vitro* assay (Figure 5).

### *i + 7* stapled peptides impair Gag processing without inhibiting virus release

**Figure 6:**
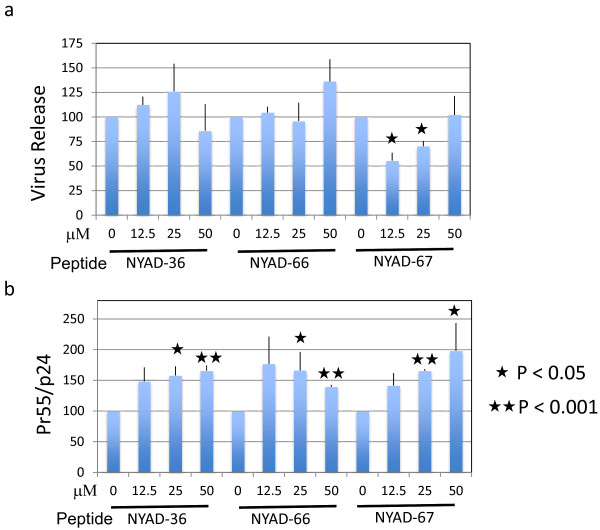
***i + 7 *stapled peptides have no effect on HIV-1 release, but impair Gag processing. (a)** 293T cells were transfected with pNL4-3 and 6 h after transfection treated with indicated concentrations of NYAD-36, -66, and -67 for 16–20 h. Cells were metabolically labeled with [^35^S]Met/Cys for 2 h. Cells were lysed and virions were collected by ultracentrifugation. Cell and virus lysates were immunoprecipitated with HIV-Ig and subjected to SDS-PAGE. Protein band intensities were quantified by phosphorimager analysis, and HIV-1 release was calculated as the amount of virion-associated p24 relative to total (cell- plus virion-associated) Gag. **(b)** Accumulation of unprocessed Gag in cells was measured by calculating the ratio of Pr55^Gag^ to p24 in cells. P values were calculated by Student’s t-test, with P < 0.05 considered significant. N = 3, ± SD.

We have shown previously that NYAD-1, an *i,i + 4* stapled peptide derived from CAI, inhibited HIV-1 release in a dose-dependent manner. This inhibition was specific to HIV-1 as the release of another lentivirus, equine infectious anemia virus (EIAV), was not inhibited [24]. In this study we investigated whether *i,i + 7* stapled CAI-derived peptides have any effect on virus release. 293T cells were transfected with the full-length HIV-1 molecular clone pNL4-3, treated with stapled peptides for 16–20 h, and metabolically labeled with [^35^S]Met/Cys. The cell- and virus-associated proteins were immunoprecipitated with HIV-Ig. Unlike NYAD-1, these analogs showed little to no inhibition of HIV-1 release (Figure 6a; Additional file 1: Figure S3). However, as we had observed previously with NYAD-1 [24], treatment of cells with each of the three stapled peptides (NYAD-36, -66, and -67) led to a modest accumulation of cell-associated Pr55^Gag^ (Figure 6b; Additional file 1: Figure S3).

### HIV-1 particles produced in the presence of stapled peptides display impaired infectivity

**Figure 7:**
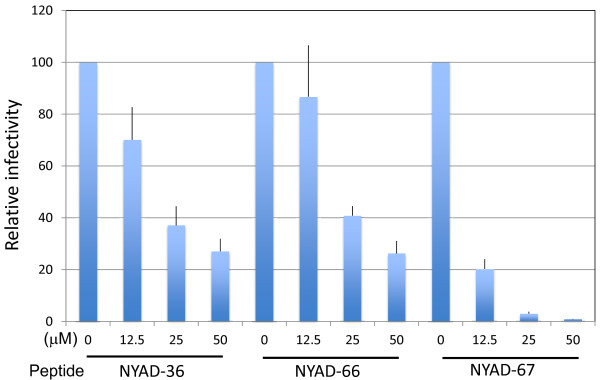
**HIV-1 particles produced in the presence of stapled peptides show defects in infectivity.** Six hours posttransfection with pNL4-3, 293T cells were treated with indicated concentrations of NYAD-36, -66, and -67 and virions were collected after 2 days. RT-normalized virus stocks were used to infect TZM-bl cells for 2 h in a total volume of 100 μl (the concentration of peptides from producer cells was diluted ~20-fold). Two days after infection, cells were washed, lysed and assayed for luciferase activity. N = 8, ± SD.

Proper Gag processing is required for particle maturation and infectivity [37,38]. Since Pr55^Gag^ processing was reduced in the presence of *i,i + 7* stapled peptides, we investigated whether the infectivity of virions produced in the presence of these peptides was inhibited. Six hours posttransfection, 293T cells were treated with the indicated concentrations of the peptides for two days and virus supernatants were collected and monitored for RT activity. The indicator cell line TZM-bl was infected with RT-normalized virus for 2 h. As shown in Figure 7, the virions produced from cells treated with 50 μM NYAD-67 showed a two-log reduction in infectivity. The infectivity defect was more moderate (four-fold) at similar concentrations of NYAD-36 and -66.

### Selection of i + 7 stapled peptide-resistant virus

**Figure 8:**
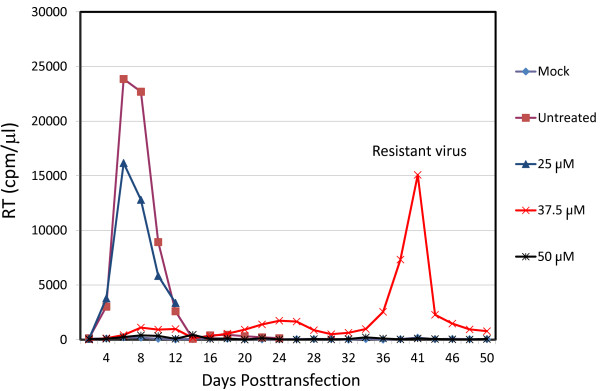
**Selection for NYAD-36-resistant virus.** Jurkat cells were transfected with pNL4-3 and cultured in the presence of 25, 37.5 and 50 μM NYAD-36. Cells were split every 2 days and HIV-1 replication was monitored by RT activity. The results are representative of at least two independent experiments.

The data presented above indicate that stapled peptides disrupt Pr55^Gag^ processing and reduce particle infectivity. To elucidate the mechanism(s) by which the infectivity of virions produced in the presence of stapled peptides was reduced, we selected for viral resistance. We used NYAD-36, a moderate inhibitor among the three stapled peptides, for the selection experiments. The Jurkat T-cell line was transfected with pNL4-3, and virus replication was monitored by RT activity in the presence of 25, 37.5, and 50 μM NYAD-36. In the absence of NYAD-36 and in the presence of 25 μM peptide, virus replication peaked approximately six days posttransfection. However, in the presence of 37.5 μM NYAD-36 virus replication was significantly delayed, with peak RT levels occurring at six weeks posttransfection (Figure 8). At 50 μM NYAD-36 no replication was detected even three months posttransfection (Figure 8, data not shown). To determine whether the delayed HIV-1 replication was due to emergence of resistance, we repassaged the virus collected on day 41 in the presence of 37.5 μM NYAD-36 in parallel with virus obtained from untreated cultures. We observed that the virus obtained from NYAD-36-treated cells showed comparable replication kinetics in the absence and presence of 37.5 μM NYAD-36, whereas the replication of WT virus was delayed under these conditions (data not shown). This analysis demonstrated that a resistant virus had emerged in the cultures treated with 37.5 μM NYAD-36.

### Identification and characterization of NYAD-36-resistant mutants

To identify the change(s) responsible for NYAD-36 resistance, viral genomic DNA was purified from infected cells at the peak of RT activity. We amplified the *gag* and *env* coding regions by PCR, and sequenced the PCR products. In two independent experiments, two mutations, V120Q and A327P, were identified in the gp120-coding portion of the *env* gene whereas no changes were observed in the *gag* gene. V120Q is located in constant region 1(C1); A327P is at the base of the V3 loop. The appearance of mutations in *env* is intriguing as the stapled peptides are designed to bind CA. We next sought to confirm that the selected mutations confer resistance to the stapled peptides by constructing pNL4-3 derivatives containing both V120Q and A327P substitutions.

**Figure 9:**
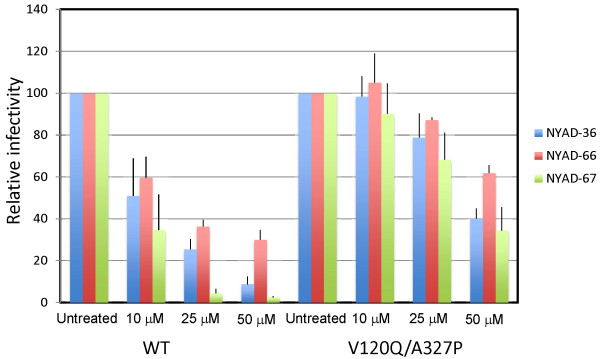
**Mutations in the HIV-1 Env confer resistance to stapled peptides in single-cycle infectivity assay.** Stapled peptides were added to TZM-bl cells at indicated concentrations during the 2 h infection for WT and Env mutant (V120Q/A327P) viruses. Two days after infection cells were washed and luciferase activity was measured as in Figure 7. N = 3, ± SD.

Since the NYAD-36-resistance mutations are located in *env*, we carried out single-cycle infectivity assays. TZM-bl cells were infected with WT and mutant HIV-1 in the absence of peptides or in the presence of NYAD-36, -66, or -67, and luciferase activity was measured two days post-infection. As shown above (Figure 9), the infectivity of the WT was reduced in a dose-dependent manner by all three peptides; at a 50 μM concentration virus infectivity was reduced >10-fold and >40-fold in the presence of NYAD-36 and NYAD-67, respectively, and around 3-fold with NYAD-66 at this concentration (Figure 9). In contrast, the infectivity of the V120Q/A327P double mutant was reduced by only ~2.5-fold at 50 μM NYAD-36 and NYAD-67, and less than 2-fold in the presence of NYAD-66 (Figure 9). These data demonstrate that the V120Q/A327P mutations in gp120 confer a marked degree of resistance to the peptides NYAD-36, -66, and -67 in single-cycle infectivity assays. We also tested the infectivity of WT and V120Q/A327P mutant virus in the presence of the previously described [24] peptide NYAD-1. The infectivity of both WT and the V120Q/A327P mutant was reduced to a similar extent (~10-fold) in the presence of NYAD-1 (Additional file 1: Figure S4) indicating that the gp120 mutations do not confer resistance to NYAD-1.

**Figure 10:**
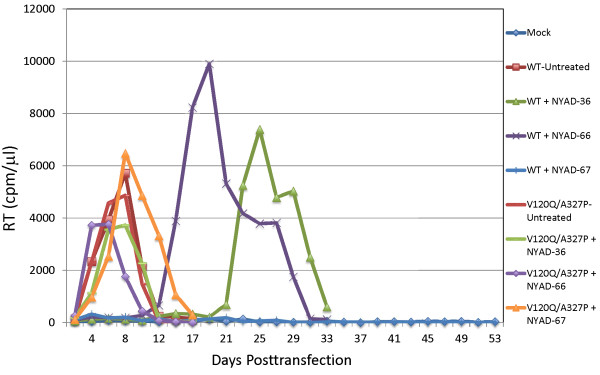
**Env mutant V120Q/A327P replicates in the presence of stapled peptides.** Replication of WT and V120Q/A327P Env mutant was carried in the presence of 37.5 μM stapled peptides as described in the Figure 8 legend. The results are representative of at least two independent experiments.

We next examined whether the V120Q/A327P Env mutant is resistant to the peptides in spreading virus replication assays. Jurkat cells were transfected with WT or the V120Q/A327P Env mutant derivative, and replication was monitored in the absence or presence of 37.5 μM stapled peptides. In the absence of peptide, virus replication kinetics of the V120Q/A327P mutant were comparable to those of the WT. Replication of the WT was significantly delayed (NYAD-36 or -66) or was completely blocked (NYAD-67) in the presence of peptide; in contrast, none of the three peptides had a significant effect on the replication of the V120Q/A327P mutant (Figure 10). These results demonstrate that the V120Q/A327P mutant is resistant to these stapled peptides in spreading infections in the Jurkat T-cell line.

**Figure 11:**
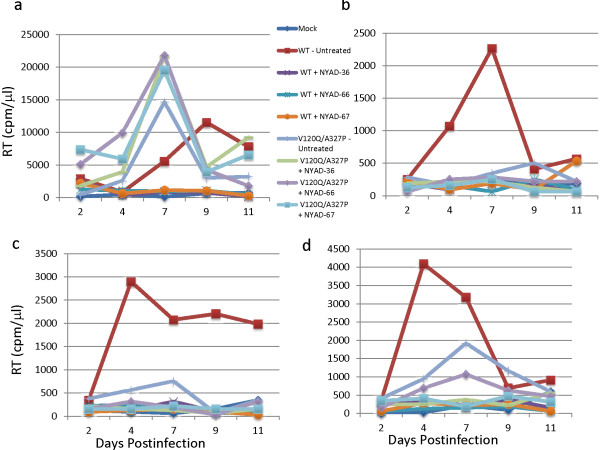
**The Env mutant V120Q/A327P is replication defective in PBMC.** Jurkat T-cell line **(a)** and PBMCs **(b-d)** were infected with RT-normalized WT- and V120Q/A327P mutant viruses and cultured in the presence of 30 uM stapled peptides and replication was monitored as described in the Figure 8 legend. The replication of HIV-1 is shown in PBMCs from three different donors **(b-d)**.

To evaluate the effects of the peptides on replication of WT and the V120Q/A327P mutant in primary human cells, we used these viruses to infect peripheral blood mononuclear cells (PBMC) from three different donors and propagated the infected cultures in the presence of 30 μM peptide. We included the Jurkat T-cell line as a control. As we observed in Jurkat cells (Figures 10 and 11a), the NYAD-36, -66, and -67 peptides strongly inhibited replication of WT NL4-3 in PBMC (Figure 11b-d). However, in contrast to the results obtained in Jurkat cells, we found that replication of the V120Q/A327P mutant was severely attenuated in the PBMC from all three donors. This result is not entirely surprising, given the high degree of conservation of gp120 residues V120 and A327 (see Discussion). Although the low level of V120Q/A327P virus replication made the antiviral activity of the peptides difficult to assess in PBMC, it appeared that NYAD-36, -66, and -67 showed some antiviral activity against the V120Q/A327P mutant in this cell type.

### The *i,i + 7* stapled peptides retain some inhibitory activity against V120Q/A327P-mutant virions produced in the presence of peptide

**Figure 12:**
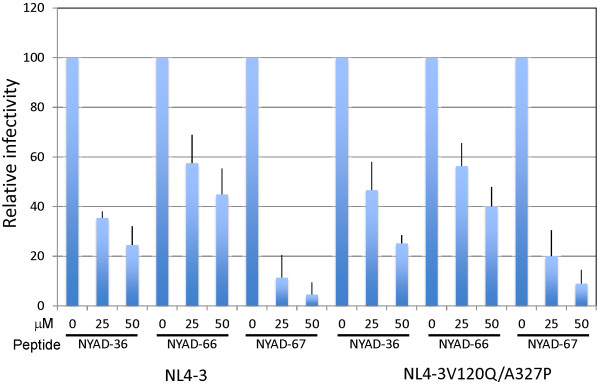
**V120Q/A327P mutant virions produced in the presence of stapled peptides were defective in single-cycle infectivity assay.** 293T cells were transfected with WT and V120Q/A327P molecular clones, six hours posttransfection cells were treated with indicated concentrations of NYAD-36, -66, and -67 and virions were collected after 2 days as in Figure 7. RT-normalized virus stocks were used to infect TZM-bl cells and infectivity was measured as described in legend to Figure 7. N = 6, ± SD.

The data presented thus far suggest that the *i,i + 7* stapled peptides exhibit inhibitory activity directed against both Gag and Env. The anti-CA inhibition can be elicited during the assembly phase of the virus replication cycle if peptide is present in the virus-producer cell (see Figure 7). This CA-based effect should not be affected by the mutations in Env that induce resistance in a spreading infection in Jurkat cells. To test this hypothesis, we analyzed the infectivity of Env mutant virions produced in the presence of stapled peptides. We transfected 293T cells with WT and V120Q/A327P molecular clones, and six hours later treated with varying concentrations of stapled peptides. Virus-containing supernatants were harvested, normalized for RT activity, and used to infect TZM-bl cells. As shown in Figure 12, both WT and V120Q/A327P virions produced from NYAD-36, -66, and -67-treated cells showed similar reductions in particle infectivity. These results suggest that although resistance mutations map to Env, and confer resistance to the peptides both in single-cycle infectivity assays when peptide is present only at the time of infection and in replication assays, these stapled peptides are still able to exert an inhibitory activity when the V120Q/A327P mutant is produced in 293T cells in the presence of peptides. These results support the hypothesis that *i,i + 7* stapled peptides exert a dual effect by inhibiting both Env and CA. Mutations in Env overcome the defects imposed during entry, but do not provide resistance to the CA-related defects.

### VSV-G-pseudotyped HIV-1 particles prepared in the presence of *i,i + 7* stapled peptides are defective in single-cycle infectivity

**Figure 13:**
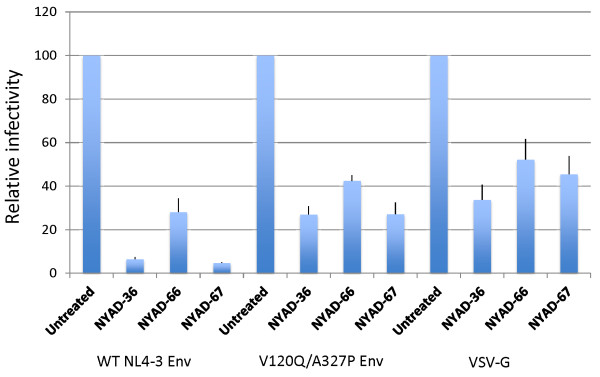
**VSV-G pseudotyped HIV-1 particles prepared in the presence of stapled peptides are also defective in single-cycle infectivity.** 293T cells were cotransfected with an Env-defective pNL4-3 derivative (pNL4-3/KFS) and vectors expressing WT or V120Q/A327P Env, or VSV-G expression vector. Six hours after transfection cells were treated with 50 μM of stapled peptides, and virions were collected after 2 days. TZM-bl cells were infected with RT-normalized virus and infectivity was measured as described in legends to Figure 7. N = 6, ± SD.

To investigate the potential dual-targeting of the peptides, we examined whether they are able to inhibit the infectivity of VSV-G-pseudotyped particles, which should be resistant to an Env-dependent entry defect but not to a CA-based effect. We also included as controls particles pseudotyped with WT or V120Q/A327P HIV-1 Env. 293T cells were cotransfected with an Env-defective pNL4-3 derivative (pNL4-3/KFS) and vectors expressing WT or V120Q/A327P Env, or VSV-G. Six hours after transfection, cells were treated with 50 μM stapled peptide and virions were collected after two days and used to infect TZBM-bl cells. The results indicated that the infectivity of all of the pseudotyped particles was reduced by the peptides. Particles bearing WT HIV-1 Env were more significantly inhibited than those bearing either the V120Q/A327P Env mutant or VSV-G (Figure 13). These results are consistent with inhibitory activity that is both Env-dependent and Env-independent.

### Pseudotyping with VSV-G reverses the ability of the peptides to inhibit virus infectivity when present during infection

**Figure 14:**
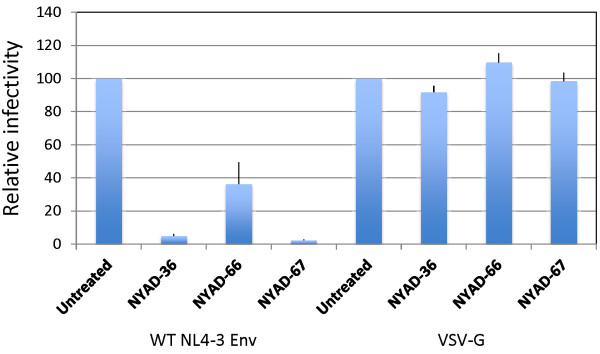
**Infectivity defects in the presence of stapled peptides are reversed by VSV-G pseudotyping.** HIV-1 virions were pseudotyped with VSV-G as described in Figure 13. RT-normalized WT HIV-1 Env- and VSV-G-pseudotyped virions were used to infect TZM-bl cells in the presence of 50 μM of stapled peptides for 2 h. Two days after infection luciferase activity was xmeasured as in Figure 7. N = 6, ± SD.

As shown above (Figure 10), *i,i + 7* stapled peptides inhibit the infectivity of HIV-1 when present at the time of infection and during virus production. The Env mutant V120Q/A327P is largely resistant to the peptide-induced entry block but remains susceptible to peptide-induced defects imposed during virus production in 293T cells. These results suggest that VSV-G pseudotyping could potentially overcome the defect elicited by the peptides at the time of infection. Indeed, we observed that particles bearing VSV-G were not inhibited by the presence of peptide during the 2 hour infection period (Figure 14). In contrast, as shown earlier, the infectivity of particles bearing HIV-1 Env was severely inhibited under these conditions.

### *i,i + 7* stapled peptides potently inhibit a large panel of HIV-1 Env-pseudotyped reference viruses

Because of the high variability between HIV-1 Env glycoproteins from different HIV-1 isolates, we tested the peptides against a large panel (total 36) of diverse HIV-1 subtype A, A/D, A2/D, AG, B, C and D reference Env-pseudotyped viruses in a single-cycle assay. It has been reported that these pseudoviruses exhibit a neutralization phenotype that is typical of most primary HIV-1 isolates and they include a wide spectrum of genetic, antigenic and geographic diversity [39,40]. The results (Table 2) indicate that all three stapled peptides tested against the pseudoviruses in single-cycle assay showed broad anti-HIV-1 activity independent of the viral subtype. Most significantly, NYAD-67 was the most active and showed consistent potent antiviral activity with an IC_50_ ranging from 59 nM to 2.6 μM. These stapled peptides showed no toxicity in TZM-bl cells at the highest dose tested (100 μg/ml).

**Table 2:** **Antiviral activity (IC**_**50**_**) of *i & i + 7 *** **stapled peptides against HIV-1 subtype A, A/D, A2/D, AG, B, C and D ENV pseudotyped reference viruses**

**NIH #**	**ENV clone**	**Subtype**	**NYAD-36**	**NYAD-66**	**NYAD-67**
			**IC**_**50 **_**(μM)**	**IC**_**50 **_**(μM)**	**IC**_**50 **_**(μM)**
11887	Q259env.w6	A	3.5 ± 0.1	0.97 ± 0.04	0.22 ± 0.02
11891	QF495.23M.ENV.A3	A	3.9 ± 0.3	3.1 ± 0.26	1.6 ± 0.06
11892	QF495.23M.ENV.B2	A	4 ± 0.7	1.8 ± 0.2	0.9 ± 0.1
11894	QG984.21M.ENV.A3	A	8.4 ± 0.5	1.9 ± 0.2	0.059 ± 0.01
11896	QH343.21M.ENV.A10	A	3.6 ± 0.2	0.38 ± 0.03	0.56 ± 0.08
11897	QH343.21M.ENV.B5	A	2 ± 0.25	2.2 ± 0.1	0.59 ± 0.09
11901	QA790.204I.ENV.A4	A/D	1.3 ± 0.3	0.63 ± 0.06	0.22 ± 0.02
11903	QA790.204I.ENV.C8	A/D	2 ± 0.3	1.2 ± 0.2	0.36 ± 0.06
11904	QA790.204I.ENV.E2	A/D	1.9 ± 0.1	1 ± 0.1	0.47 ± 0.04
11905	QG393.60M.ENV.A1	A2/D	6.5 ± 1.2	1.1 ± 0.22	1.1 ± 0.11
11906	QG393.60M.ENV.B7	A2D	~10.8	9 ± 0.9	2.1 ± 0.2
11907	QG393.60M.ENV.B8	A2/D	7.9 ± 0.6	10.4 ± 0.6	1.8 ± 0.06
11591	CRF02_AG Clone 211	AG	9.4 ± 0.2	15.3 ± 0.7	1.8 ± 0.05
11595	CRF02-AG Clone 251	AG	4.1 ± 0.3	1.1 ± 0.08	0.95 ± 0.03
11599	CRF02-AG Clone 257	AG	3.9 ± 0.17	2.2 ± 0.26	0.62 ± 0.02
11604	CRF02_AG Clone 271	AG	2 ± 0.05	3 ± 0.2	0.15 ± 0.01
11022	PVO, clone 4 (SVPB11)	B	1.6 ± 0.2	0.43 ± 0.08	0.26 ± 0.006
11023	TRO, Clone 11 (SVPB12)	B	7.7 ± 0.8	>14	2.3 ± 0.2
11035	pREJO4541 clone 67 (SVPB16)	B	5.7 ± 0.6	8.2 ± 0.2	0.84 ± 0.03
11036	pRHPA4259 clone 7 (SVPB14)	B	3.1 ± 0.3	0.66 ± 0.17	0.42 ± 0.1
11037	pTHRO4156 clone 18 (SVPB15)	B	3.6 ± 0.32	1.7 ± 0.15	0.48 ± 0.01
11038	pCAAN5342 clone A2 (SVPB19)	B	4.8 ± 0.6	0.77 ± 0.13	0.47 ± 0.06
11058	SC422661, clone B (SVPB8)	B	7.1 ± 0.5	3.8 ± 0.2	1.8 ± 0.1
11507	HIV-25925-2, clone 22	C	6.8 ± 0.3	1.3 ± 0.15	1 ± 0.13
11508	HIV-26191-2, clone 48	C	4.5 ± 0.3	1.5 ± 0.4	2.6 ± 0.5
11307	Du172.17	C	3.5 ± 0.15	1.8 ± 0.1	0.47 ± 0.02
11308	Du422.1	C	2.3 ± 0.25	1.3 ± 0.2	1.1 ± 0.11
11310	ZM214M.PL15	C	1.9 ± 0.1	1 ± 0.06	0.39 ± 0.02
11313	ZM53M.PB12	C	1.7 ± 0.05	1.1 ± 0.06	1.6 ± 0.2
11314	ZM109F.PB4	C	3.3 ± 0.2	3.2 ± 0.2	0.7 ± 0.1
11317	CAP210.2.00.E8	C	3.6 ± 0.05	2.5 ± 0.1	0.9 ± 0.09
11911	QA013.70I.ENV.H1	D	3.5 ± 0.2	1.4 ± 0.3	0.48 ± 0.06
11912	QA013.70I.ENV.M12	D	3.4 ± 0.1	2 ± 0.2	0.45 ± 0.02
11913	QA465.59M.ENV.A1	D	1.8 ± 0.3	1.2 ± 0.1	2 ± 0.3
11914	QA465.59M.ENV.D1	D	0.93 ± 0.1	0.98 ± 0.02	1.2 ± 0.06
11916	QD435.100M.ENV.B5	D	10.1 ± 1.9	2 ± 0.1	0.3 ± 0.03

### Mapping of the binding site of the stapled peptides on gp120

The series of experiments described above indicate that the *i,i + 7* stapled peptides target viral entry in an Env-dependent manner. We therefore wanted to identify the possible binding site of these peptides on gp120. First, we used an optical biosensor approach to measure the affinity of NYAD-36, NYAD-66 and NYAD-67 to immobilize full-length Yu2 gp120 or a V3-loop-deleted variant Yu2 gp120ΔV3. NYAD-36, NYAD-66 and NYAD-67 tightly bind to the full-length Yu2gp120 with *K*_*d*_ of 1.7 μM, 2.6 μM and 1.3 μM, respectively (Additional file 1: Figure S5a-c). The deletion of V3 on Yu2Gp120 impaired the binding of NYAD-36 and NYAD-67 by more than 5-fold.

We also used ITC to measure the binding affinity of the stapled peptides to full-length cyclic V3 loop peptide. Only NYAD-36 and NYAD-67 were observed to bind to the cyclic V3 loop peptide with *K*_d_ of 0.35 ± 0.03 μM and 0.99 ± 0.04 μM, respectively (Additional file 1: Figure S6a-d). The reverse titration resulted in similar binding constants. To further map the binding domain on V3, we measured the binding affinity of NYAD-36 and NYAD-67 with a series of overlapping V3 loop peptides. The results indicate that neither N-terminal nor C-terminal V3 loop peptides bind to NYAD-36 or NYAD-67. However, the tip of V3 loop retains binding to NYAD-36 and to NYAD-67 with *K*_d_ of 15.06 ± 3.78 μM and 8.92 ± 1.80 μM, respectively (Additional file 1: Figure S7a-c). These binding data also suggest that the whole conformation of V3 loop is important for the interaction.

## Discussion

We previously reported that an *i,i + 4* stapled peptide, NYAD-1, penetrates cells and disrupts virus assembly in both *in vitro* and cell-based assays. Significantly, NYAD-1 specifically inhibited HIV-1 release without affecting the release of another lentivirus, EIAV [24]. In addition, direct binding analyses by ITC and NMR confirmed that NYAD-1 binds to the CTD of CA. These findings motivated us to expand the scope of our drug discovery effort on hydrocarbon-stapled peptides as assembly and maturation inhibitors to an *i,i + 7* series of stapled peptides. This approach allowed us to explore residues not available in the *i,i + 4* series for enhancing binding and antiviral activity. Both these stapling techniques generally convert linear and non-cell-penetrating peptides to α–helical and cell-penetrating peptides. Despite the fact that this new series of stapled peptides showed α-helical secondary structures, their ability to penetrate cells was somewhat less efficient compared to *i,i + 4* peptides, such as NYAD-1, which has recently been confirmed to penetrate cells through lipid bilayers even at low concentrations [41]. The detailed study of cell penetration by Sun et al. [41] indicates that NYAD-1 primarily binds to the polar-apolar interface with its helix parallel to the lipid bilayer, which helps stretching and thinning the membrane as was observed with other helical antimicrobial peptides such as magainin and melittin. In the case of *i,i + 7* stapled peptides reported here, their lesser ability to penetrate cells may be attributed to their net charge; these peptides are acidic, whereas NYAD-1 is neutral at physiological pH. Similar poor cell permeability of an *i,i + 7* stapled peptide, SAH-p53-4, that binds the E3 ubiquitin ligase hDM2, was reported by Bernal et al. in 2007 [42]. This peptide has a net charge of -2 at physiological pH. However, this group used structure-guided optimization to overcome the problem of cell permeability by modifying the acidic residues, Glu and Asp, which were not located at the binding interface, to Gln and Asn, respectively. These modifications had no adverse effect on the binding affinity between SAH-p53-4 and hDM2. However, when we replaced two Glu and one Asp residues in NYAD-36 these peptides became more toxic (NYAD-46, -58 in Table 1). One probable explanation for this failure is that we used the NMR structure of NYAD-1 [32], in the absence of any structure of *i,i + 7* stapled peptides used in this study, in the structure-guided modifications. It is possible that NYAD-46 differs from NYAD-1 in terms of its structure and CTD binding features.

The *i,i + 7* stapled peptides, which showed moderate cell penetration, were designed based on the original linear peptide CAI, which binds to the CA CTD and inhibited virus assembly *in vitro*. Therefore, we wanted to confirm whether these peptides bind to the CA CTD and inhibit *in vitro* particle assembly. Indeed, we observed that they not only bind to the CA CTD but also the binding affinity (*K*_d_) of one of the most potent *i,i + 7* stapled peptides, NYAD-67 (2.63 μM), was similar to that of NYAD-1 (2.00 μM) in ITC experiments. However, the binding affinity of NYAD-36 was about 5-fold lower. HSQC NMR experiments confirmed tight binding of these peptides to mutant CA (W184A/M185A). Interestingly, impaired Gag processing was observed when 293T cells transfected with a full-length HIV-1 molecular clone were treated with *i,i + 7* stapled peptides. Furthermore, when HIV-1 particles were produced in the presence of these stapled peptides they showed impaired infectivity (Figure 7). The above studies and biophysical data demonstrate that these stapled peptides interact with the CA CTD and diminish particle infectivity.

To identify the target of these stapled peptides, resistant viral variants were selected in the presence of one of the moderately active *i,i + 7* stapled peptides, NYAD-36. Unexpectedly, no mutation associated with resistance was identified in the CA region; rather, resistance mutations mapped to two residues in gp120: V120Q in the C1 region and A327P at the base of the V3 loop. An NL4-3 derivative encoding the V120Q/A327P double mutant showed substantial resistance to NYAD-36, -66 and -67 compared to the WT NL4-3 virus. Inspection of gp120 sequences from 170 different HIV-1 isolates aligned in the Los Alamos National Laboratory HIV sequence database (http://www.hiv.lanl.gov/content/index) revealed >95% and >98% conservation of Val (V) at position 120 and Ala (A) at position 327, respectively. These findings explain the broad anti-HIV-1 activity of these peptides, especially NYAD-67, which showed consistently potent activity against a large panel of viruses from the different clades we tested, including representatives from clade C that is responsible for more than 50% of infections worldwide. It is noteworthy that in all selection experiments these two mutations arose together. It is currently not clear what role each of these mutations plays in conferring resistance; the x-ray structure of gp120 (pdb:1G9M) shows that V120 and A327 are >27Å apart but they are both on the CD4-binding interface of gp120 suggesting that they may play a significant role in HIV-1 entry. V120 is located near the C-terminus of C1, which is in close proximity to two of the CD4-binding site residues (P124 and V127 in HIV-1 HXB2 and NL4-3) near the V1 loop. A327P is located at the stem of V3 loop, which has been shown to play a critical role in HIV-1 coreceptor binding [43,44]. Suphaphiphat et al. reported that an A327G mutation had very little effect on CCR5 binding but it substantially reduced HIV-1 infectivity [45]. Indeed, in their study, a major effect on infectivity was observed for mutations of most of the residues in the C-terminal portion of the V3 loop stem. We speculate that the A327P mutation arose in response to direct binding of the negatively charged stapled peptides, NYAD-36, -66 and -67, to the highly positively charged RKSIRIQRGPGR (in NL4-3 virus) motif around the crown of the V3 loop. Direct binding analysis by SPR using full-length Yu2 gp120 or a V3-loop-deleted variant Yu2 gp120ΔV3 supports this hypothesis (Additional file 1: Figure S5). The importance of both the crown and stem region of V3 has been reported in the binding of gp120 to CCR5 in mediating virus entry [46]. A recent report by Yokoyama et al. showed that the recombinant virus with a gp120 V3 loop carrying a net charge of +3, but not with net charge of +7, was resistant to neutralization by CD4 binding site targeted antibodies [47]. We [48,49] and others [50,51] have reported the effect of negatively charged compounds that bind to the V3 loop and elicit antiviral activity. The negatively charged compounds and peptides may alter the net charge of the V3 loop and thereby elicit similar effect. Recently, Yuan et al. showed by molecular dynamic (MD) simulations of wild type (WT) and maraviroc-resistant gp120 V3 loop that the mutations in the stem region might affect the structural dynamics of the loop, and it was suggested that changes in loop dynamics and/or orientation might confer resistance to maraviroc [52] by inhibiting interactions with cellular coreceptors. Taken together the results suggest that these peptides act as bi-functional antiviral agents by targeting gp120 to inhibit virus entry and CA to disrupt CA function. Similar bi-functional effects of betulinic acid derivatives have been reported [53]. Chemical modification of the side chains at C-3 and C-28 in betulinic acid conferred maturation inhibitor and entry inhibitor activity, respectively. When both side chain modifications were incorporated in the new derivatives they became bi-functional and the potency was enhanced by at least 20 times compared to one of the most potent maturation inhibitors and clinical candidates, bevirimat or the entry inhibitor lead compound IC9564.

We note that while the gp120 mutations selected during propagation of NL4-3 in the Jurkat T-cell line conferred nearly complete resistance to the peptides in virus replication assays in Jurkat cells (Figure 10), some inhibitory peptide activity remained when the mutant virus was produced in 293T cells in the presence of peptide and infectivity assayed in the HeLa-based TZM-bl indicator cell line (Figure 12). This was also the case when the Env mutant was propagated in PBMC (Figure 11). We hypothesize that the observation of full resistance in Jurkat and partial resistance in the single-cycle assays and PBMC replication assays may reflect differential uptake or activity of the peptides in different cell types; as one would expect, the mutations in V3 are sufficient to largely counteract the entry defect conferred by the peptides but would not affect their CA-targeted antiviral activity. This is also consistent with the observation that the previously described peptide NYAD-1 [24] is active against both WT and the Env mutant virus in single-cycle infectivity assays.

## Conclusion

In conclusion, the in-depth mechanistic study of *i,i + 7* peptides reported here suggests that the design of stapled peptides is critical and it might have significant influence on the ability of the stapled peptides to target a single step or multiple steps in HIV-1 life cycle. The study presented here is expected to be useful in optimizing these lead peptides to more active and selective inhibitors.

## Methods

### Cells, virus and compounds

The MT-2 T-cell line [obtained through the NIH AIDS Research and Reference Reagent Program (ARP) from Dr. D. Richman] and the Sup-T1 T-cell line (obtained from Dr. James Hoxie) [54] were grown in RPMI 1640 medium (Invitrogen) supplemented with 10% fetal bovine serum (FBS), penicillin and streptomycin. TZM-bl cells (a HeLa cell line derivative that expresses CD4, CXCR4 and CCR5 and expresses luciferase and ß-galactosidase under control of the HIV-1 promoter, obtained through the NIH ARP from Dr. John C. Kappes, Dr. Xiaoyun Wu and Tranzyme Inc) [55,56], HEK 293T cells (ATCC) were grown in Dulbecco's modified Eagle's medium (DMEM) (Invitrogen) supplemented with 10% FBS, penicillin and streptomycin. PBMCs were isolated and cultured as previously described [57]. The HIV-1 molecular clones HIV-1_IIIB_ and pNL4-3 were obtained through the NIH ARP from Dr. R. Gallo and M. Martin, respectively. Plasmid pNL4-3.Luc.R-.E- (Dr. N. Landau) [58,59] and pSG3Δ^env^ DNA (Drs. J.C. Kappes and X. Wu) [56,60], were obtained through the NIH ARP. Env expression vector pHXB2-env (X4) was obtained through the NIH ARP from Dr. K. Page and Dr. D. Littman [61] and Env expression vector pSVIIIenv-ADA was kindly provided by Dr. JG Sodroski [62]. Clones representing the standard HIV panels A, A/D, A2/D and D were obtained through the NIH ARP from Dr. Julie Overbaugh [63,64]. The panel of subtype A/G Env clones were also obtained through the NIH ARP from Drs. Ellenberger, D., Li, B., Callahan, M., and Butera, S. [65]. The HIV-1 Env panel of standard reference subtype B Env clones were obtained through the NIH ARP from Drs. David Montefiori, Feng Gao and Ming Li (PVO, clone 4 (SVPB11), TRO, Clone 11 (SVPB12), SC422661, clone B (SVPB8)); from Drs. B.H. Hahn and J.F. Salazar-Gonzalez (pREJO4541 clone 67 (SVPB16), pRHPA4259 clone 7 (SVPB14)); from Drs. B.H. Hahn and D.L. Kothe (pTHRO4156 clone 18 (SVPB15), pCAAN5342 clone A2 (SVPB19)) [39,56,60]. The subtype C HIV-1 reference panel of Env clones were also obtained through the NIH ARP from Drs. D. Montefiori, F. Gao, S. Abdool Karim and G. Ramjee (Du172.17); from Drs. D. Montefiori, F. Gao, C. Williamson and S. Abdool Karim (Du422.1), from Drs. B. H. Hahn, Y. Li and J.F. Salazar-Gonzalez (ZM214M.PL15); from Drs. E. Hunter and C. Derdeyn (ZM53M.PB12 and ZM109F.PB4); from Drs. L. Morris, K. Mlisana and D. Montefiori, (CAP210.2.00.E8) [39,66,67]. The subtype C Indian Env clones (HIV-25925-2, clone 22 and HIV-26191-2, clone 48) were obtained from Drs. R. Paranjape, S. Kulkarni and D. Montefiori [65].

Full-length V3 peptide (Cat# 1840), cyclic V3 peptide (Cat# 1837) and 15-mer overlapping V3 peptides (Cat# 1832, 6282, 6283, 6284, 6285, 6287, 6288, 6289, 6290 and 6291, for amino acid sequence see HIV-1 Subtype B (MN) Env Peptides - Complete Set Cat# 6451) were obtained through the NIH ARP.

### Molecular cloning, protein expression and purification

pET14b or pET28a plasmids encode Gag-derived proteins from the HIV-1 NL4-3 strain. The full-length gag expression vector was subcloned from HIV-1 NL4-3 strain. The CA coding region was obtained by PCR amplification and was inserted into the pET28a vector. The C-CA (CTD) DNA fragment was provided by Drs. Ming Luo and Peter E Prevelige Jr [68]. The C-CA (CTD) DNA fragment was subcloned into the pET14b vector. The CTD mutant (W184A/M185A) was generated from pET14b-C-CA using Stratagene's QuikChange® Site-Directed Mutagenesis Kit following the manufacture’s protocol. The corresponding proteins (including ^15^N or ^15^N/^13^C labeled mutant C-CA and C-CA) were expressed and purified as described previously [68]. Protein concentrations were determined with the A_280_ molar extinction coefficients of 2,980 M^-1^cm^-1^(CTD, mutant), 8,480 M^-1^cm^-1^(CTD), 33,460 M^-1^cm^-1^(CA) and 64,400 M^-1^cm^-1^ (Gag), respectively.

### Synthesis of the *i,i + 7* stapled peptides

The peptides were synthesized manually by Fmoc solid phase synthesis using Rink amide MBHA resin (0.3-0.4 mmol/g). For the normal amino acids, the couplings were performed with four-fold excess of amino acids. Fmoc-amino acids were activated using the ratio of Fmoc-amino acid:HBTU:HOBt:DIEA, 1:1:1:2. For (*S*)-*N*-Fmoc-2-(4′-pentenyl)alanine (Fmoc-*S*_5_-OH, Okeanos Tech Co. Ltd) and (*R*)-*N*-Fmoc-2-(7′-octenyl)-alanine (Fmoc-*R*_*8*_-OH, Okeanos Tech Co. Ltd), the double coupling was performed with two-fold excess of amino acid which was activated with DIC:HOAt (1:1) or HCTU. For peptide olefin metathesis, the peptide resin with N-terminal protected by Fmoc group was treated with degassed 1, 2 dichloroethane containing Bis(tricyclohexylphosphine)-benzylidine ruthenium (IV) dichloride (10 mM) at room temperature for two hours and the reaction was repeated once for completion. After de-Fmoc, the resin bound peptide was cleaved using standard protocols (95% TFA, 2.5% water, 2.5% TIS). The cleaved peptide was purified by RP-HPLC using 0.1% (v/v) TFA/water and 0.1% (v/v) TFA/acetonitrile and its identity was confirmed using electrospray mass spectroscopy.

### Pseudovirus preparation

Pseudoviruses capable of single-cycle infection were obtained by transfecting HEK 293T cells as previously described [17]. Briefly, 5x10^6^ HEK 293T cells were seeded in a T75 flask and 24 h later, transfected in 15ml medium with a mixture of 10 μg of an env-deleted proviral backbone plasmid, pNL4-3.Luc.R-.E- DNA or pSG3Δ^env^ DNA and 10 μg of an Env expression vector using FuGENE 6 (Roche) following manufacturer’s instructions. Pseudovirus-containing supernatants were collected 2 days after transfection, filtered and stored in aliquots at -80°C.

### Virus assembly and release assays

293T cells, plated at 3 × 10^5^ cells/well in 12 well plates, were transfected with the HIV-1 molecular clone pNL4-3 [69]. Six h after transfection, cells were treated with indicated concentrations of NYAD-36, -66, and -67 for 16–20 h. One day posttransfection, cells were metabolically labeled with [^35^S]Met/Cys for 2 h. The labeled virions were pelleted in an ultracentrifuge, and the virus and cell lysates were immunoprecipitated with pooled sera from HIV-1-infected patients (HIV-Ig, obtained from the ARP) and subjected to SDS-PAGE. Protein band intensities were quantified by phosphorimager analysis, and virus release was calculated as the amount of virion-associated Gag as a fraction of total (cell- plus virion-associated) Gag synthesized during the metabolic labeling period. Accumulation of Pr55^Gag^ in cells was measured by calculating the ratio of Pr55^Gag^ to p24 (CA).

### Single-cycle infection assay

The inhibitory activity of *i,i + 7* stapled peptides NYAD-36, NYAD-66 and NYAD-67 was measured on HIV-1 pseudotyped viruses expressing Env from the panel of standard reference subtype A, A/D, A2/D, AG, B, C and D. Pseudoviruses were obtained by transfecting HEK 293T cells with a mixture of an Env-deleted backbone proviral plasmid pSG3^Δenv^ and an Env expression vector DNA. Briefly, for the neutralization assay 100 μl of TZM-bl cells at 1 × 10^5^ cells/ml were added to the wells of a 96-well tissue culture plate and cultured at 37°C overnight. 50 μl of a staple peptide at graded concentrations was mixed with 50 μl of the HIV-1 pseudovirus at about 100 TCID_50_ (50% tissue culture infectious dose). After incubation at 37°C for 30 min, the mixture was added to the cells and incubated at 37°C for 3 days. Cells were washed 2 times with PBS and lysed with 50 μl of cell culture lysis reagent. 20 μl of lysates were transferred to a white 96-well plate and mixed with 100 ul of luciferase assay reagent. The luciferase activity was immediately measured as above to calculate IC_50_ (Luciferase Assay System, Promega). The luciferase activity was immediately measured with a Tecan Infinite M100 reader (Tecan, USA) and the IC_50_ values were calculated by the GraphPad Prism software (GraphPad Software, Inc., USA).

TZM-bl cells were seeded at 1 × 10^4^ cells/well in 96 well plates; one day later cells were infected with reverse-transcriptase (RT)-normalized virus stocks for 2 h. Stapled peptides (NYAD-1, -36, -66, -67) were added during the 2 h infection period at indicated concentrations. Two days postinfection, cells were washed with PBS, lysed in luciferase lysis buffer (Promega) and luciferase activity was measured with luciferase assay substrate (Promega). In experiments where virus-producing cells were treated with the peptides, six-hour posttransfection 293T cells were treated with indicated concentrations of NYAD-36, -66, or -67, and virus supernatant was collected after 2 days. Env pseudotyped viruses were generated by cotransfecting 293T cells with an Env-defective pNL4-3 derivative (pNL4-3/KFS) [70] and HIV-1 Env expression vector pIIINL4env [71,72], pIIINL4env-V120Q/A327P, or VSV-G expression vector pHCMV-G [73].

### Multi-cycle infection assay

The inhibitory activity of *i,i + 7* stapled peptides on infection by laboratory-adapted HIV-1_IIIB_ strain was determined as previously described [24]. In brief, 10^4^ MT-2 cells were infected with HIV-1_IIIB_ at 100 TCID_50_ (0.01MOI) in the presence or absence of test compounds at graded concentrations and incubated overnight. The culture supernatants were then replaced with fresh media. On the fourth day post-infection, 100 μl of culture supernatants were collected from each well, mixed with equal volume of 5% Triton X-100 and tested for p24 antigen by sandwich ELISA. The percentage of inhibition of p24 production and IC_50_ values were calculated with the GraphPad Prism software (GraphPad Software Inc.).

### Determination of cytotoxicity

#### In MT-2 cells

Cytotoxicity of stapled peptides in MT-2 cells was measured with the XTT (Sodium 3’-[1-phenylamino-carbonyl]-3,4-tetrazolium]-bis[4-methoxy-6-nitro]benzenesulfonic acid hydrate) method as previously described [24]. Briefly, 100 μl of a stapled peptide at graded concentrations was added to an equal volume of cells (10^5^cells/ml) in 96-well plates followed by incubation at 37°C for 4 days. Following addition of XTT (PolySciences, Inc.), the soluble intracellular formazan was quantitated colorimetrically at 450 nm 4 h later. The percent of cytotoxicity and the CC_50_ values were calculated with the GraphPad Prism software (GraphPad Software Inc.).

#### In TZM-bl cells

The cytotoxicity of stapled peptides in TZM-bl cells was also measured by the colorimetric method using XTT. Briefly, 100 μl of a compound at graded concentrations was added to equal volume of cells (10^5^/ml) in wells of 96-well plates followed by incubation at 37°C for 3 days. Following addition of XTT the soluble intracellular formazan was quantitated as described above.

### Selection and characterization of NYAD-resistant viruses and virus replication assays in the Jurkat T-cell line and PBMCs

NYAD-36-resistant viral isolates were selected by prolonged serial passage of wild-type (WT) NL4-3 in Jurkat cells in the presence of 25, 37.5 and 50 μM NYAD-36. Virus replication during the selection process was monitored by RT activity as previously described [74]. Virus supernatants and cell pellets were collected on the days of peak RT activity. RT-normalized viruses were used to infect fresh Jurkat cells, and virus replication was examined as above to confirm acquisition of NYAD-36 resistance. To identify the mutations conferring resistance to NYAD-36, genomic DNA was extracted from cells on the day of peak RT activity using a whole blood DNA purification kit (Qiagen). The entire Gag and Env coding region was amplified by PCR and sequenced. Putative resistance-conferring mutations were introduced into the Env expression vector pIIINL4env by site-directed mutagenesis (Stratagene) using mutagenic oligonucleotides. Following sequence confirmation, the EcoRI-NheI fragment was cloned back into the WT pNL4-3 to generate a molecular clone containing the Env mutations, which were confirmed by sequencing. PBMCs isolated from healthy donors were activated by phytohemagglutinin (PHA) and were infected with RT-normalized WT- and V120Q/A327P mutant viruses and replication was carried out in the presence of 30 M NYAD-36, -66, or -67. Virus supernatant was collected every 2 or 3 days, and RT activity was monitored as above.

### NMR studies

A pET11a plasmid containing DNA for a monomeric HIV-1 CA, p24-W184A/M185A, with a C-terminal His tag was transformed into Rosetta 2 (DE3) cells (EMD) for protein expression. The His-tag was not removed prior to NMR and turbidity studies. ^15^N isotopically labeled protein was expressed in a minimal media solution supplemented with ^15^NH_4_Cl. Protein overexpression was induced with 1mM IPTG at an OD_600_ of 0.6 overnight at 37°C. After expression, the cells were harvested by centrifugation and lysed using a microfluidizer (Microfluedics) using lysis buffer containing 50 mM NaPO_4_ [pH 7.4] 5 mM BME and 10 mM imidazole. Lysed cells were then centrifuged and the soluble fraction was applied to a cobalt resin (Thermo Scientific) for purification. The resin was washed with lysis buffer and CA was eluted with lysis buffer containing 250 mM imidazole. Pure protein was dialyzed in NMR buffer containing 50 mM NaOAc [pH 5.5] 5 mM DTT with 10%D_2_O.

The NMR-based titrations were collected at 35°C on a Bruker AVANCE III 600 MHz equipped with a cryogenic probe. Staple peptides were dissolved in 100% DMSO and titrated into the monomeric HIV-1 CA, p24-W184A/M185A, in a buffer containing 50 mM NaOAc [pH 5.5] 5 mM DTT with 10%D_2_O. Increasing molar ratios of staple peptides were added into the protein and chemical shift perturbations were monitored.

### Circular dichroism spectroscopy

Circular dichroism (CD) spectra were obtained on an Aviv model 62DS CD spectrometer (Aviv, Lakewood, NJ) at 25°C using the standard measurement parameters in 1× PBS in the presence of 1–20% (v/v) acetonitrile at a final concentration of 100 μM. In all samples, the final concentrations of peptides and salt were always the same, and the spectra were corrected by subtracting the CD spectrum of the appropriate reference solvent.

### Confocal microscopy

For the cell penetration study, 293T cells were seeded in 4-well chamber plates and incubated at 37°C with 5 μΜ of FAM-conjugated peptides for 20 h in medium containing serum. After three washes with 1X PBS, live cells were examined and imaged under a Zeiss LSM510 laser scanning confocal microscope (Zeiss).

### FACS Analysis of FAM-conjugated Peptide Treated Cells

297-T cells were grown in RPMI 1640 (Gibco), 10% fetal bovine serum, 100 U/ml penicillin, 100 μg/ml streptomycin, 2 mM glutamine, 50 mM Hepes pH 7, and 50 μM ß-mercaptoethanol. 293-T cells (6x10^5^/well) in 2 ml serum-free media were treated with FAM-conjugated NYAD-1, NYAD-66, NYAD-67, NYAD-36 and NYAD-41. The final concentration of the corresponding FAM-conjugated peptides is 2 μM, 4 μM or 8 μM. After 4 hours (data not shown) or 20 hours incubation with the FAM-conjugated peptides at 37°C, the treated cells were washed twice with 1x PBS. After a treatment with 0.25% Trypsin-EDTA (GIBCO) for 30 min at 37°C and two washes with 1× PBS, the cells were subjected to FACS analysis*.* The data were analyzed by using Flowjo Software.

### Electron microscopy to study inhibition of *in vitro* assembly by peptides

In vitro assembly experiments were set up as described [9,75-77] with minor modification. We used 50 mM Na_2_HPO_4_, pH 8.0 as the dialysis buffer. The buffer used for assembly studies also contained 0.1 ~ 2 M of NaCl. 500-Da-MWCO dialysis tubes (Spectra/Por) were used for the peptide dialysis. Briefly, stock proteins were adjusted to the appropriate concentration (25 μΜ for Gag protein or 50 μΜ for CA protein) with the Na_2_HPO_4_ buffer at pH 8.0. After incubation with varied doses of NYAD-36, NYAD-66 and NYAD-67 for 30 min at 4°C, the samples were dialyzed overnight in Na_2_HPO_4_ buffer at pH 8.0 containing 100 mM NaCl at 4°C. Negative staining was used to check the assembly. Carbon-coated copper grids (200 mesh size; EM Sciences) were treated with 20 μl of poly-L-lysine (1 mg/ml; Sigma) for 2 min. 20 μl of reaction solution was placed onto the grid for 2 min. Spotted grids were then stained with 30 μl of uranyl acetate solution for 2 min. Excess stain was removed, and grids were air-dried. Specimens were examined with a TECNAI G^2^ electron microscope (FEI, OR, USA).

### I*n vitro* CA assembly

Wild-type HIV-1 CA was overexpressed and purified as previously described [10]. Pure protein was dialyzed in NMR buffer containing 50 mM NaPO_4_ [pH 8.0]. Turbidity assays were carried out in triplicate using a Beckman spectrophotometer at a wavelength of 350 nm to monitor HIV-1 in vitro assembly conducted at ambient temperatures [10]. Protein samples were prepared in an assembly buffer [50 mM NaPO_4_ pH 8.0]. In vitro assembly reactions were prepared with 250 μl of assembly buffer containing 60 μM protein. To catalyze CA assembly 250 μl of an assembly buffer containing 5M NaCl was added. The solution was mixed by agitation and transferred a cuvette for a total delay of 20 seconds. Assembly was monitored every 10 sec for the first min and every min thereafter for a total 10 min. Assembly reactions with stapled peptides were incubated in the assembly buffer for 10 min and then centrifuged for 1 min to separate any insoluble fractions before catalyzing assembly.

### Isothermal titration calorimetry (ITC)

The binding affinity between CTD (W184A/M185A) and NYAD-36, NYAD-66 or NYAD-67 was measured by ITC at 30°C using a Microcal titration calorimeter (MicroCal VP-ITC). Both CTD (W184A/M185A) protein and stapled peptides were exhaustively dialyzed against 25 mM sodium phosphate buffer (pH 7.3) prior to experimental measurements. In a typical experiment, the ITC injection syringe was loaded with 625 μM CTD (W184A/M185A) protein, dissolved in the dialysis buffer. The calorimetric cell (ca. 1.4 ml active volume) initially contained only 25 μM stapled peptide in the identical dialysis buffer. Typically titrations consisted of 27 injections of 10 μl into the calorimetric cell, with 240-s equilibration between injections. Both forward and reverse titrations were used to confirm the interactions between V3 loop peptide and stapled peptides.

For forward titration, 90 μM full-length V3 peptide, full-length cyclic V3 peptide or 90 μM 15-mer V3 peptides were titrated into 4.5 μM of stapled or linear peptides. Reference measurements were performed by titrating 90 μM full-length V3 peptide, full-length cyclic V3 peptide or 15-mer V3 peptide into buffer without peptide. For reverse titration, 90 μM stapled peptide was titrated into 4.5 μM full-length V3 peptide, full-length cyclic V3 peptide or 90 μM 15-mer V3 peptide. Reference measurements were performed by titrating 90 μM stapled peptide into buffer without peptide.

### Surface plasmon resonance (SPR)

The binding kinetics and affinity of stapled peptides to HIV-1 Yu2gp120 (kindly provided by Dr. Peter Kwong, Vaccine Research Center, NIH) were analyzed by SPR (BIAcore 3000, Piscataway, NJ). The Yu2gp120 or Yu2gp120 protein with V3 loop deletion (Yu2gp120ΔV3) was covalently immobilized to a CM5 sensor chip via amine groups using the amine coupling kit (BIAcore) in 10 mM sodium acetate buffer at pH 5.0. The immobilization level of gp120 reached about 1,500 response units (RU).

For the binding affinity assay, the stapled peptides were prepared as a 10 mM stock solution in 100% DMSO. Immediately prior to analysis, the compounds were diluted with Dulbecco's PBS to a final DMSO concentration of 3%. Experiments were run at 30 μl/min in running buffer (Dulbecco's PBS containing 3% DMSO). The stapled peptides at different concentrations were injected over the target protein (gp120) and reference surfaces for 2 min, followed by a 5 min dissociation period. The surface was regenerated with 6.25 mM NaOH and 50 μl/min for 30 s.

The binding kinetic parameters were evaluated with BIA-Evaluation software (BIAcore 3000, Piscataway, NJ) in which all data sets were fit to a simple 1:1 (Langmuir) binding model including a term for mass transport. The data selection of the binding and dissociation curves for fitting was based on the instruction manual of the BIAcore 3000 instrument, in which 3–5 s of data at association and dissociation start were eliminated.

## Competing interests

The authors declare that they have no competing interests.

## Authors’ contributions

AKD, EOF and MFS designed experiments, analyzed and prepared the manuscript. HZ designed, performed in vitro assembly assay and biophysical studies by SPR and ITC. AC advised on analysis and interpretation of biophysical study data. FC performed antiviral assays and analyzed the data. AAW designed, performed and analyzed the infectivity and selection experiments. PYM, MM, PB performed the NMR and the in vitro assembly experiments and analyzed the data. DS assisted in antiviral screening. XT and SL prepared all stapled peptides. All authors read and approved the final manuscript.

## Supplementary Material

Additional file 1: Figure S1 Cell penetration of (a) NYAD-41, NYAD-36, NYAD-66 and (b) NYAD-67 and NYAD-1 in 293T cells. **Figure S2.** Isothermal titration calorimetric (ITC) analyses of the interaction betweenCTDM184A/W185A and NYAD-D36, NYAD-66, NYAD-67 or NYAD-1. **Figure S3.***i+7* stapled peptides have no effect on HIV-1 release, but impair Gag processing. **Figure S4.** The Env mutant V120Q/A327P is not resistance to NYAD-1. **Figure S5.** Kinetic analysis of the interaction between gp120 and (a) NYAD-36, (b) NYAD-66 or (c) NYAD-67 by SPR. **Figure S6.** Isothermal titration calorimetric (ITC) analyses of the interaction between fulllength cyclic V3 loop peptide and NYAD-41, NYAD-36, NYAD-66, NYAD67 or NYAD-1. **Figure S7.** Isothermal titration calorimetric (ITC) analyses of the interaction between 15-mer V3 tip peptide and NYAD-36, NYAD-66 or NYAD-67. Click here for file
