# Supplementary material for: Dual-acting stapled peptides target both HIV-1 entry and assembly
Source: Retrovirology. 2013 Nov 15;10:136. doi: 10.1186/1742-4690-10-136 (PMC3842668; doi:10.1186/1742-4690-10-136)
Supplement: Additional file 1: Figure S1 — Cell penetration of (a) NYAD-41, NYAD-36, NYAD-66 and (b) NYAD-67 and NYAD-1 in 293T cells. Figure S2. Isothermal titration calorimetric (ITC) analyses of the interaction betweenCTDM184A/W185A and NYAD-D36, NYAD-66, NYAD-67 or NYAD-1. Figure S3.i+7 stapled peptides have no effect on HIV-1 release, but impair Gag processing. Figure S4. The Env mutant V120Q/A327P is not resistance to NYAD-1. Figure S5. Kinetic analysis of the interaction between gp120 and (a) NYAD-36, (b) NYAD-66 or (c) NYAD-67 by SPR. Figure S6. Isothermal titration calorimetric (ITC) analyses of the interaction between fulllength cyclic V3 loop peptide and NYAD-41, NYAD-36, NYAD-66, NYAD67 or NYAD-1. Figure S7. Isothermal titration calorimetric (ITC) analyses of the interaction between 15-mer V3 tip peptide and NYAD-36, NYAD-66 or NYAD-67. [file 1742-4690-10-136-S1.pdf]

Additional Files

Fig. S1. Cell penetration of (a) NYAD-41, NYAD-36, NYAD-66 and (b) NYAD-67 and NYAD-1 in 293T cells. Representative confocal microscopy images of 293T cells incubated for 20 h at 37 °C with FAM-conjugated peptides. For each panel: left differential interference contrast (DIC) image of cells with FAM-β-Ala-conjugated peptide: center, FAM fluorescent image of the same cells with FAM-β-Ala-conjugated peptide and right, overlay of DIC and FAM fluorescent images.

Fig. S2. Isothermal titration calorimetric (ITC) analyses of the interaction between CTDM184A/W185A and NYAD-D36, NYAD-66, NYAD-67 or NYAD-1. 625 μM CTDM184A/W185A was titrated in 10 μl aliquots into (a) 20 μM NYAD-36; (b) 20 μM NYAD-66; (c) 20 μM NYAD-67 and (d) 250 μM CTDM184A/W185A into 10μM NYAD-1. Integrated binding isotherm of the titration fit to a single-site model.

Fig. S3. *i+7* stapled peptides have no effect on HIV-1 release, but impair Gag processing. 293T cells transfected with pNL4-3 were treated with indicated concentrations of NYAD-36, -66 or -67. Cells were metabolically labeled, and cell and virus lysates were immunoprecipitated and subjected to SDS-PAGE as described in the Fig. 6 legend. The fluorogram shown is representative of three independent experiments.

Fig. S4. The Env mutant V120Q/A327P is not resistance to NYAD-1. NYAD-1 was added to TZM-bl cells at 25 and 50 μM concentrations during the 2 h infection period. Two days after infection luciferase activity was measured as described in the Fig. 7 legend. N=4, ± SD.

Fig. S5. Kinetic analysis of the interaction between gp120 and (a) NYAD-36, (b) NYAD-66 or (c) NYAD-67 by SPR. Kinetic data were collected with different dosages of staple peptides against full-length gp120 or its V3 deletion mutant. Upper panel: sensogram against full-length Yu2gp120. Lower panel: sensogram against Yu2gp120 with V3 deletion (Yu2gp120ΔV3). 2-fold dilutions of staple peptides were injected and passed over the sensor chip.

Fig. S6. Isothermal titration calorimetric (ITC) analyses of the interaction between full-length cyclic V3 loop peptide and NYAD-41, NYAD-36, NYAD-66, NYAD67 or NYAD-1. 90 μM V3 loop peptide was titrated in 10 μl aliquots into (a) 4.5 μM NYAD-41, (b) NYAD-36, (c) NYAD-66, (d) NYAD-67 and (e) NYAD-1. Integrated binding isotherm of the titration fit to a single-site model

Fig. S7. Isothermal titration calorimetric (ITC) analyses of the interaction between 15-mer V3 tip peptide and NYAD-36, NYAD-66 or NYAD-67. 90 μM 15-mer V3 tip peptide was titrated in 10 μl aliquots into (a) 4.5 μM NYAD-36, (b) NYAD-66 and (c) NYAD-67. Integrated binding isotherm of the titration fit to a single-site model.

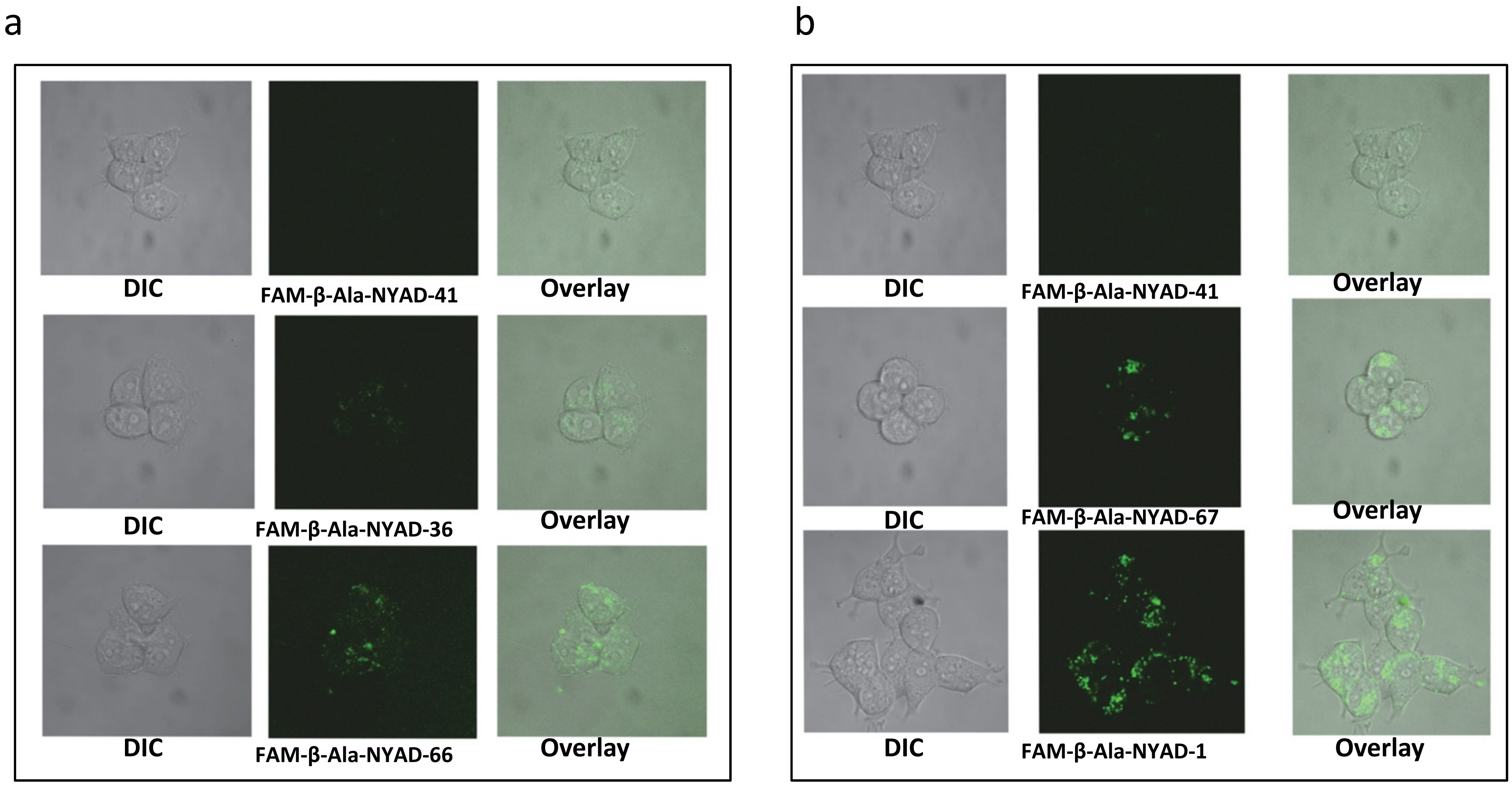
a
DIC
FAM-β-Ala-NYAD-41
Overlay
DIC
FAM-β-Ala-NYAD-36
Overlay
DIC
FAM-β-Ala-NYAD-66
Overlay
b
DIC
FAM-β-Ala-NYAD-41
Overlay
DIC
FAM-β-Ala-NYAD-67
Overlay
DIC
FAM-β-Ala-NYAD-1
Overlay

Fig. S1

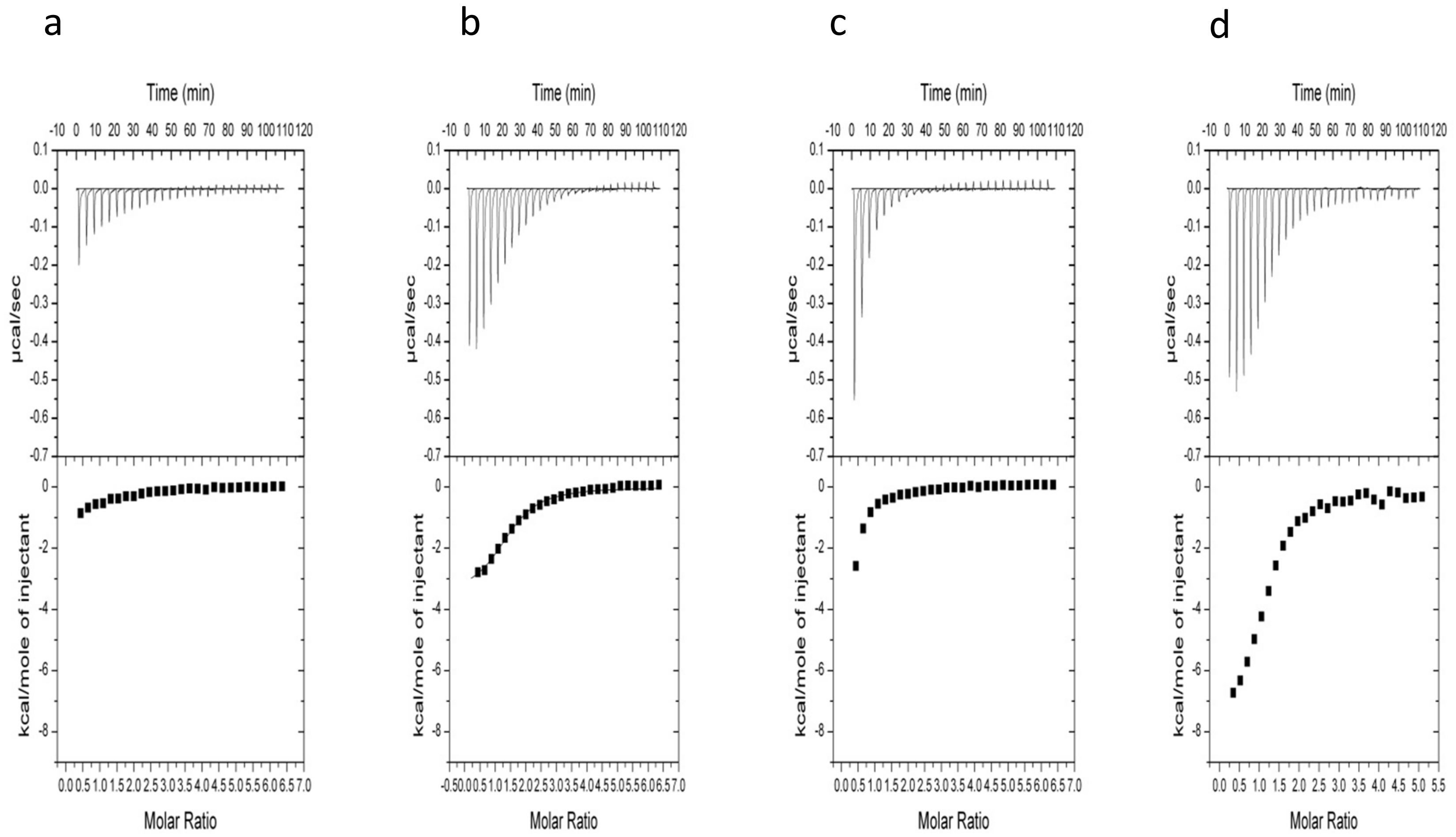

Fig. S2

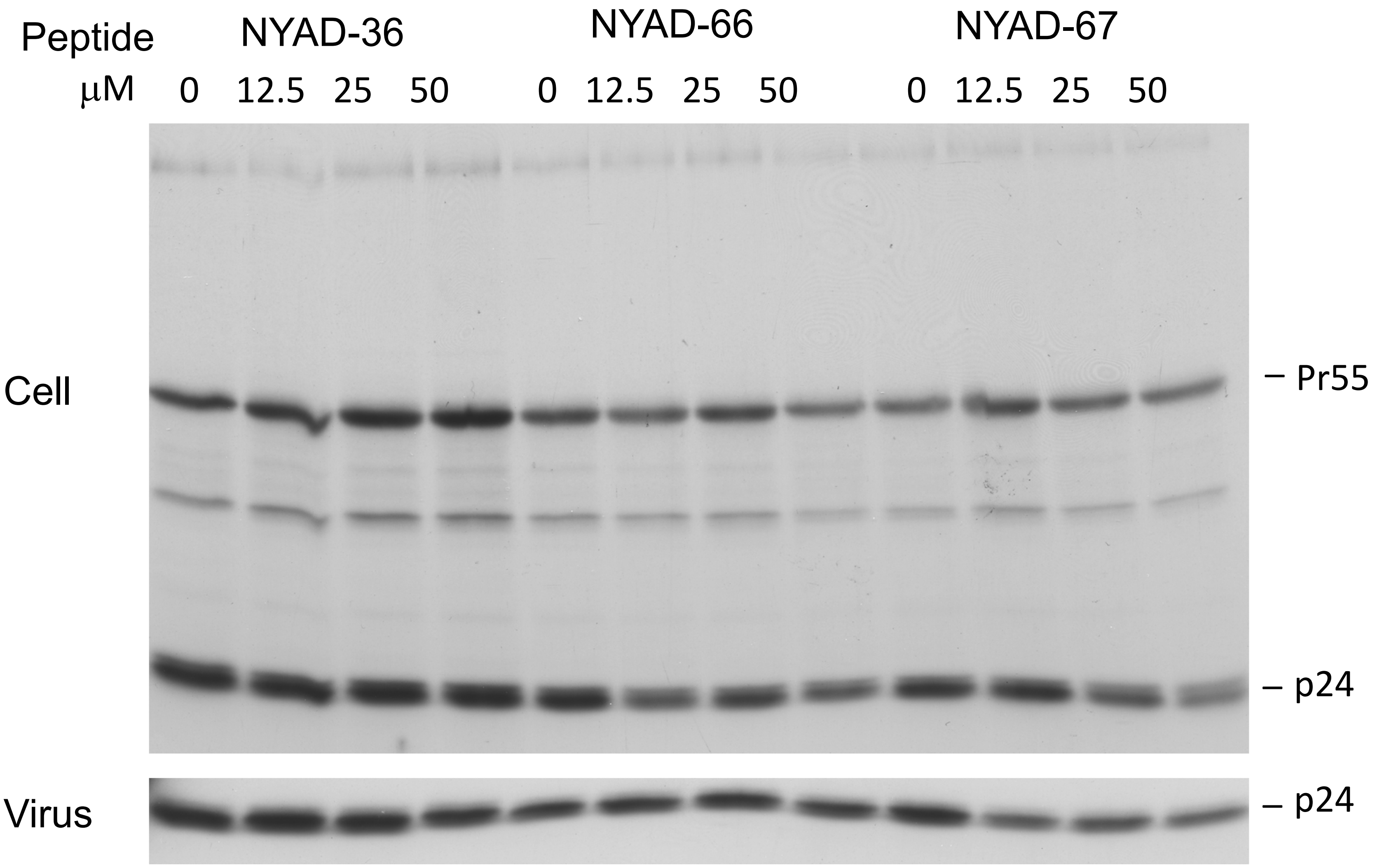
Peptide
NYAD-36
NYAD-66
NYAD-67
μM
0
12.5
25
50
0
12.5
25
50
0
12.5
25
50
Cell
Virus
Pr55
p24
p24

Fig. S3

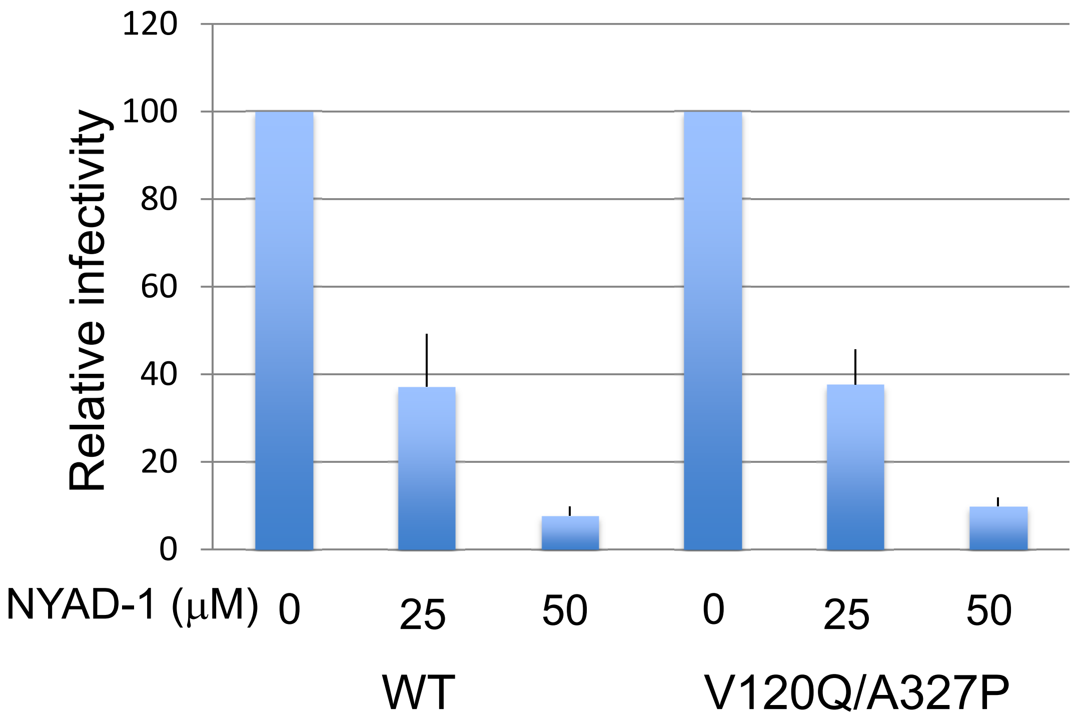

120
100
80
60
40
20
0
Relative infectivity
NYAD-1 (μM) 0 25 50 0 25 50
WT
V120Q/A327P

Fig. S4

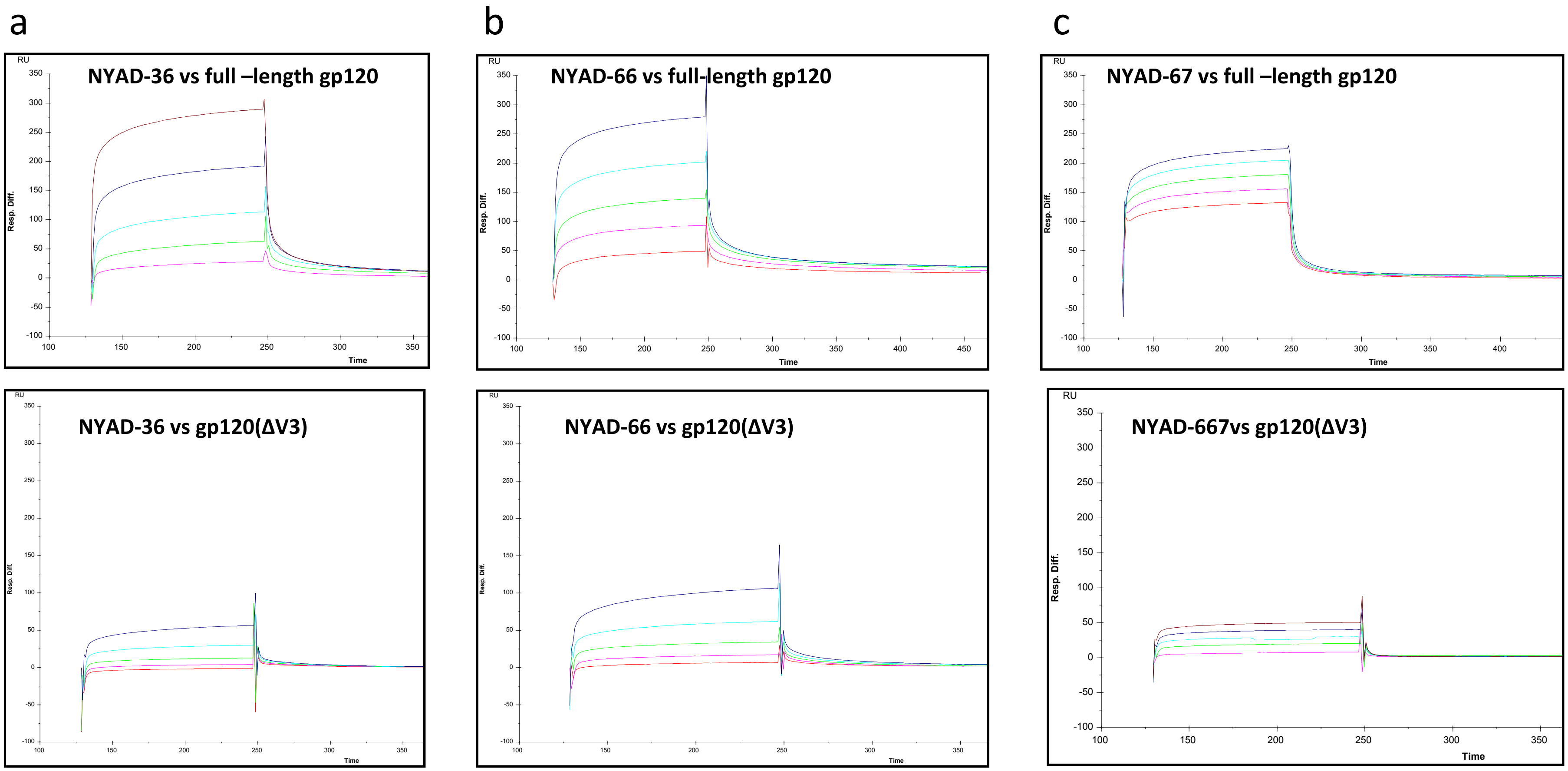
a
b
c
NYAD-36 vs full –length gp120
NYAD-66 vs full-length gp120
NYAD-67 vs full –length gp120
NYAD-36 vs gp120(ΔV3)
NYAD-66 vs gp120(ΔV3)
NYAD-667vs gp120(ΔV3)
RU
Resp. Diff.
Time

Fig. S5

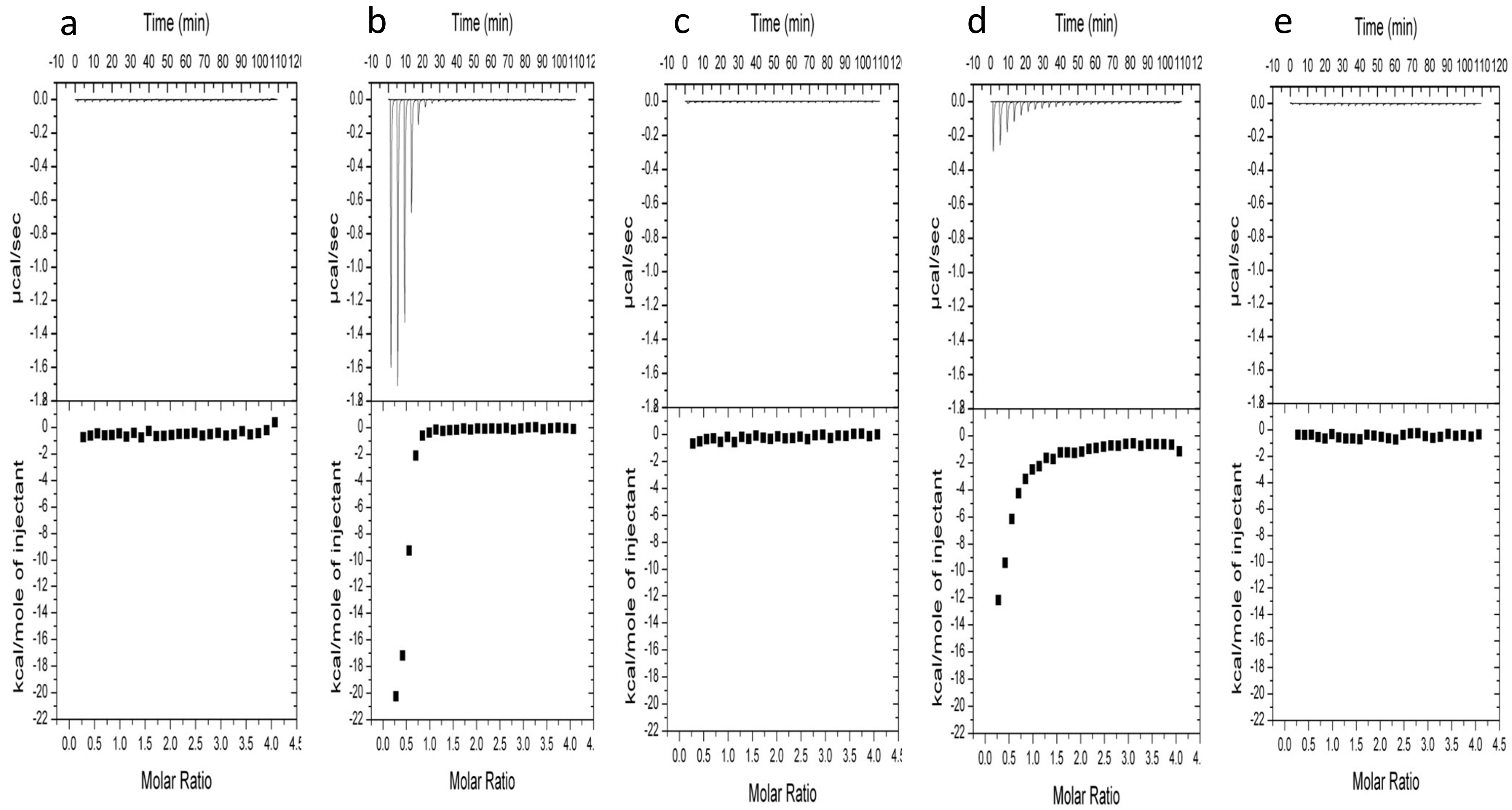

Fig. S6

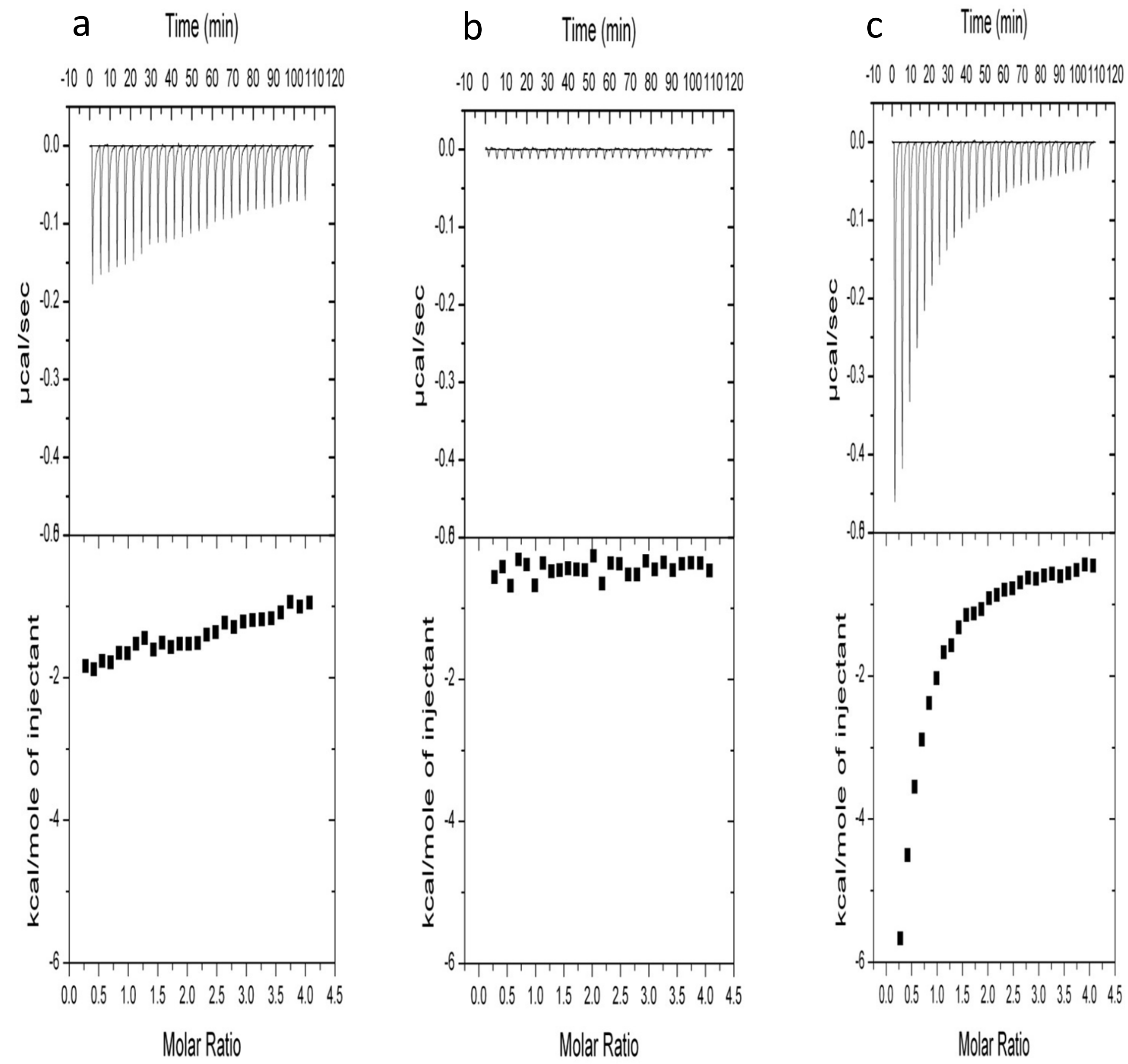
a
b
c
Time (min)
-10 0 10 20 30 40 50 60 70 80 90 100 110 120
0.0
-0.1
-0.2
-0.3
-0.4
-0.6
µcal/sec
-2
-4
-6
kcal/mole of injectant
0.0 0.5 1.0 1.5 2.0 2.5 3.0 3.5 4.0 4.5
Molar Ratio

Fig. S7
